# Regional Impacts of Unconventional Oil and Gas Operations on Ozone and Air Toxics

**DOI:** 10.1021/acs.est.6c03632

**Published:** 2026-10-19

**Authors:** Yongtao Hu, Kamal Jyoti Maji, M. Talat Odman, Jennifer Kaiser, Armistead G. Russell

**Affiliations:** School of Civil and Environmental Engineering, 1372 Georgia Institute of Technology, Atlanta, Georgia 30332, United States

**Keywords:** air quality, source attribution, population-weighted exposure, precursor emissions, benzene

## Abstract

Unconventional oil and gas development (UOGD) has expanded over the past three decades and now accounts for most of U.S. oil and natural gas production. Although prior studies link local UOGD activity to health outcomes, they provide limited evidence directly attributing ambient pollutant levels to UOGD emissions. We address this gap by quantifying UOGD impacts on ozone and air toxics using the Community Multiscale Air Quality (CMAQ) model with the Integrated Source Apportionment Method (ISAM), enabling an explicit linkage between UOGD emissions and ambient concentrations. UOGD activities produce substantial local contributions to primary air toxics and continental-scale influences on ozone. Across CONUS, UOGD increases population-weighted annual averages of maximum daily average 8-h (MDA8) ozone, 1-h benzene, 1-h nitrogen dioxide (NO_2_), and 1-h formaldehyde (HCHO) concentrations by roughly 1 ppb, 1.1 ppt, 0.035 ppb, and 0.03 ppb, respectively. Annual maximum UOGD contributions to MDA8 ozone are about half of those from other anthropogenic sources. ISAM also reveals unexpectedly large UOGD-driven ozone enhancement (up to 4–5 ppb) in the Los Angeles region from Texas emissions, a feature less evident in brute-force sensitivity tests. These findings highlight the need to further examine interstate UOGD influences on nonattainment areas and source-impact quantification methods.

## Graphical abstract

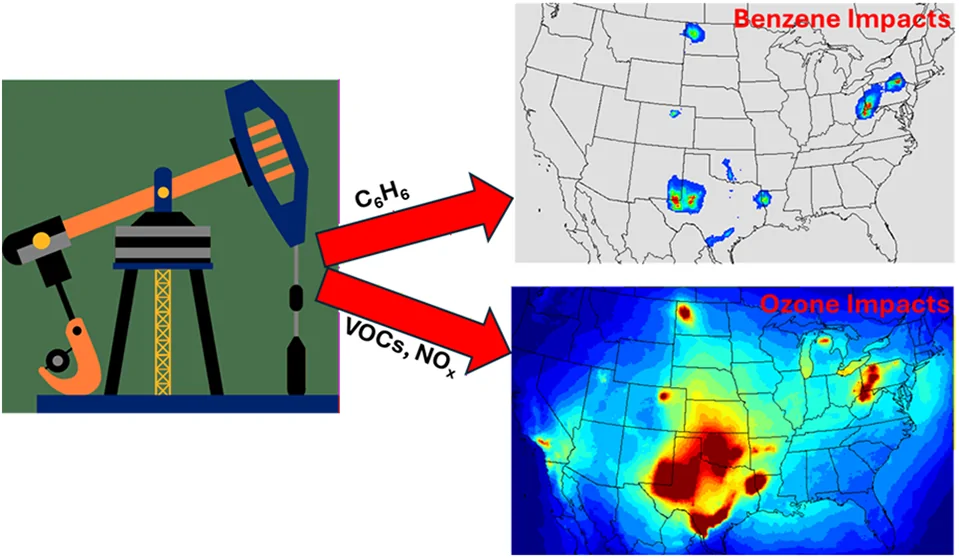

## 1 Introduction

Widespread adoption of hydraulic fracturing and horizontal drilling technologies involved in unconventional oil and gas development (UOGD) has significantly increased production of oil and gas in the United States.^1–4^ These innovations enabled access to vast shale formations across the United States (US), including the Bakken, Eagle Ford, Haynesville, Marcellus, and Wolfcamp shale plays (Figure 1).^2,5,6^ This rapid growth of UOGD activities in recent decades has brought up various environmental and health concerns, including increased exposures from elevated air pollutant levels.^7–25^

**Figure 1:**
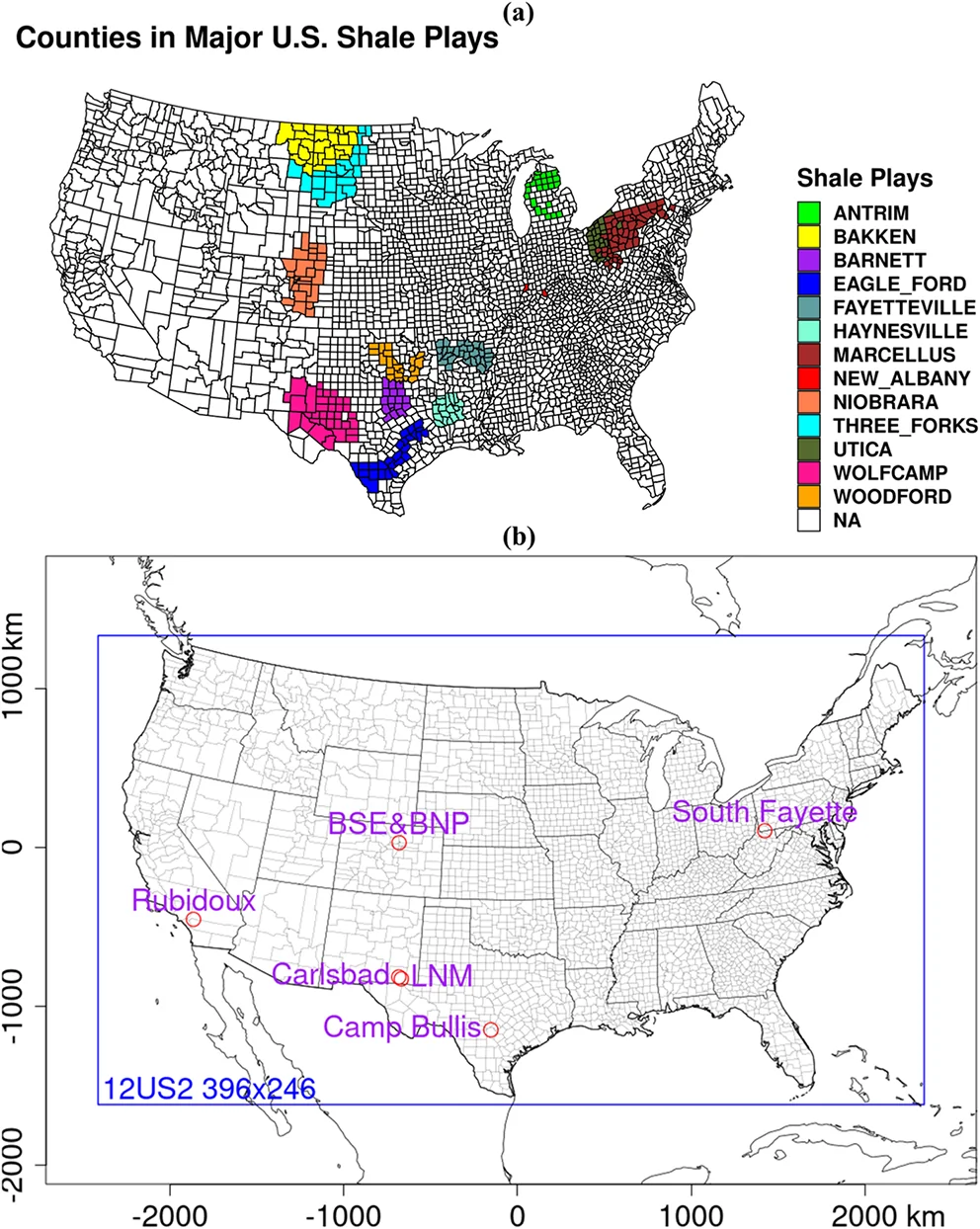
(a) Counties with major US shale plays (https://www.eia.gov/naturalgas/fieldcode/); (b) The 12-km resolution CONUS WRF-CMAQ domain. Also labeled in purple on the map are the monitoring sites with locations displayed in red circles. The sites shown are the AQS site 06-065-8001 in Rubidoux, California (Rubidoux), the AQS site 48-029-0052 in Camp Bullis, Texas (Camp Bullis), the AQS site 42-003-1008 in South Fayette, Pennsylvania (South Fayette), the AQS site 35-015-1005 in Carlsbad, Eddy County, New Mexico (Carlsbad), and the special campaign monitoring sites (see monitor information on Web site https://bouldair.com/) the Broomfield North Pecos (BNP) and Broomfield Soaring Eagle (BSE) sites near Boulder, CO (BSE&BNP) and the Loving, New Mexico site (LNM).

UOGD air pollutant emissions include those from associated vehicle traffic, power generation, equipment use, heating, pumping, drilling, spillage, fracking fluids, flowback, chemical injection, leakage, glycol dehydration, flaring, storage, loading, and transport of products, and more, leading to a mixture that is complex in composition and varying in time.^6,8,26–31^ UOGD-related emissions consist of methane, volatile organic compounds (VOCs) including air toxics (i.e., hazardous air pollutants or HAPs), and nitrogen oxides (NO_*x*_, i.e., NO+NO_2_+HONO), along with particulate matter, carbon monoxide (CO), and sulfur dioxide (SO_2_).^6,26,32–45^ Not all of the emitted chemical species have been identified.^6^ At the local scale, high acute exposures to HAPs, for example, to benzene, due to transient emissions impacting the neighboring community are of concern.^14,46,47^ At the regional scale, UOGD emissions of NO_*x*_, VOCs (including formaldehyde: HCHO), and SO_2_ contribute to secondary formation of ozone and fine particulate matter.^26^ UOGD methane emissions also contribute to climate forcing.^6^

Here we focus on local (few km) to regional-scale impacts from UOGD emissions on air quality, particularly on concentrations of ozone (O_3_) and HAPs. O_3_ is a secondary pollutant formed from its main precursors’ emissions: NO_*x*_, VOCs, and CO. Federal, state, and local regulations have resulted in decreases in total NO_*x*_ and VOC emissions by, respectively, 73 and 46% from 1990 to 2023 (https://www.epa.gov/air-trends/air-quality-national-summary, accessed July 2025), helping to decrease O_3_ concentrations nationally by ∼18%. More recent reductions have not been so rapid (e.g., ∼1% from 2010 to 2023), and there are still more than 50 Metropolitan Statistical Areas (MSAs) currently designated as nonattainment for not meeting the 2015 national ambient air quality standard (NAAQS) of 0.070 ppm for O_3_, including Denver, CO, and Los Angeles, CA (https://www.epa.gov/air-trends/air-quality-design-values), though high ozone levels are not limited to urban areas, e.g., Carlsbad Caverns likewise experience exceedances.^26,42,48^

Unlike other major sectors (for instance transportation and power generation), UOGD emissions increased in response to UOGD activities especially in those shale formation rich regions.^6,26,40^ UOGD occurs in both rural and populated areas across the United States, with spatially nonuniform developments following the distribution of shale reservoirs.^49^ Some UOGD activities overlap with the O_3_ nonattainment areas.^26,42^ Studies have shown that emissions from UOGD increase O_3_ ^26^ and that UOGD impacts on O_3_ can be significant,^50^ even though their emissions are a relatively small portion of the total emissions,^26,51–56^ e.g., a 20 ppb O_3_ impact in the Rocky Mountain National Park of Colorado.^57^ UOGD emissions can also contribute to wintertime O_3_ in remote rural communities in Wyoming and Utah.^26,58–61^ UOGD emissions can also contribute to wintertime haze.^62^

It is important for regulation purposes to assess UOGD emission impacts on air quality.^40,63^ Such air-quality impact assessments have been conducted using various source apportionment methods. Many studies have focused on source contributions to ambient speciated VOCs measured downstream of the UOGD area using receptor models or other observation-based methods,^64–68^ while some of these studies further apportioned source contributions to ozone formation potential.^69^ However, these approaches do not directly quantify the contributions of UOGD emissions to ambient pollutant concentrations and are generally unable to account for the complex role of NO_*x*_ emissions, which can either enhance or suppress ozone formation, depending on chemical and meteorological conditions. In addition, most previous source-attribution studies have focused on individual shale basins or localized UOGD-intensive regions, limiting our understanding of the broader regional impacts of UOGD emissions.^50,51,55,56,70,71^

As a result, two important scientific questions remain insufficiently addressed: (1) what are the contributions of major UOGD basins to ambient ozone and air toxics across the United States? and (2) how do these contributions vary spatially and among different UOGD regions? Addressing these questions requires a modeling framework capable of explicitly linking emissions from individual UOGD basins to ambient pollutant concentrations while accounting for atmospheric transport and nonlinear chemistry.

Chemical transport models (CTMs), such as the Comprehensive Air Quality Model with Extensions (CAMx)^72,73^ and the Community Multiscale Air Quality (CMAQ) Model,^74,75^ are the primary tools for quantitatively linking the impact of emissions on concentrations of secondary pollutants (e.g., O_3_) and are also used for other pollutants at nonlocal scales. These CTMs have been equipped with extended source apportionment tools, e.g., the Integrated Source Apportionment Method (ISAM),^76,77^ the Ozone Source Apportionment Technology (OSAT) and Particulate Source Apportionment Technology (PSAT),^73,78^ and the decoupled, direct method (DDM)^78,98–101^ that have been used to apportion contributions from different source sectors to ambient concentrations of O_3_ or PM_2.5_.^79–90^ However, source contributions explicitly from UOGD emissions have been much less studied using these numerical probing tools, instead relying more on receptor models and trajectory models^52,65,91–94^ or using a CTM with a brute-force method.^50,55,95–97^

In this study, we apply CMAQ coupled with ISAM to quantify basin-specific contributions of UOGD emissions to surface ozone and air toxics across the continental United States (CONUS) for 2023 and 2024. Unlike previous studies that relied primarily on receptor models, trajectory analyses, or perturbation-based approaches, this framework directly attributes both primary and secondary pollutants to spatially resolved UOGD emission sources within a chemically consistent modeling system, providing a nationwide assessment of UOGD contributions to ozone and air toxics from major U.S. production basins and establishing an advanced framework for linking UOGD emissions to potential population exposures of both primary and secondary air pollutants.^102^

## 2 Methods

### 2.1 Photochemical Air Quality Modeling

CMAQ (v5.4), using the CB6r5 chemical mechanism and AERO7 aerosol module, is applied to a region encompassing the contiguous United States (CONUS), southern Canada, and northern Mexico (Figure 1) with a 12 km horizontal resolution grid (12US2). Vertically, there are 34 layers extending into the lower stratosphere. Major inputs into CMAQ include emissions and meteorological fields (described in the following section).

Hourly meteorological fields were developed using the Weather Research and Forecasting (WRF) model version 4.2.1 ^103^ with NAM analyses (https://www.ncei.noaa.gov/products/weather-climate-models/north-american-mesoscale). WRF performance, evaluated against the EPA Air Quality System’s (AQS) hourly meteorological observations (https://aqs.epa.gov/aqsweb/airdata/download_files.html) at the surface, was assured for the purpose of air quality modeling (Table S1),^104,105^ with slight seasonal variations among monthly performance measures (Table S2).

CMAQ-ready sectorized emissions inputs were prepared using the EPA’s emissions modeling platforms: 2020-platform^106^ for year 2023 and 2021-platform^107^ for year 2024, with the Electricity Generation Unit (EGU) inventories updated with the year-specific Continuous Emissions Monitoring (CEM) data (https://campd.epa.gov/). These EPA emissions platforms split the US anthropogenic emissions inventories into about 20 sectors, which include estimates of emissions from UOGD operations in the “np_oilgas” (nonpoint oil and gas) and “pt_oilgas” (point oil and gas) sectors. Biomass burning emissions were not included because year-specific inventories, i.e., of agricultural burns and wildland fires (including prescribed fires and wildfires), were not available from EPA at the time. The inline BEIS4 module with BELD5 was used for the biogenic emissions. The inventories used in the SMOKE modeling within the EPA platforms include emissions for the criteria air pollutants such as NO_*x*_, VOCs, primary (directly emitted) PM_2.5_ and coarse PM, sulfur dioxides (SO_*x*_), ammonia (NH_3_) and CO. We further split the “np_oilgas” and “pt_oilgas” sectors by source category codes (SCC) and county codes into major-shale-play-specific subsectors for the purpose of source apportment modeling by using the list of “Counties in Major U.S. Shale Plays”, Appendix C of the County Code Master List (CCML, https://www.eia.gov/naturalgas/fieldcode/). CCML was developed by the Energy Information Administration (EIA), which provides standardized county names and codes for its reporting of proved reserves and production from shale reservoirs in the Form EIA-23L, i.e., “Annual Report of Domestic Oil and Natural Gas Reserves″. EIA has identified a list of 325 counties in the United States that contain proved reserves in 13 major U.S. shale plays: Antrim, Bakken, Barnett, Eagle Ford, Fayetteville, Haynesville, Marcellus, New Albany, Niobrara (aka Denver-Julesberg), Three Forks, Utica, Woodford, and Wolfcamp (aka Permian). See distributions of these shale plays in Figure 1 and their associated typical daily emissions in Figure 2 and annual emissions in Table S3. Although the major UOGD shale plays account for more than half of the oil and gas sector’s NO_*x*_ emissions and over two-thirds of its VOC emissions, their contributions to total anthropogenic emissions across CONUS are comparatively modest, representing approximately 7% of anthropogenic NO_*x*_ emissions and 13% of anthropogenic VOC emissions.

**Figure 2:**
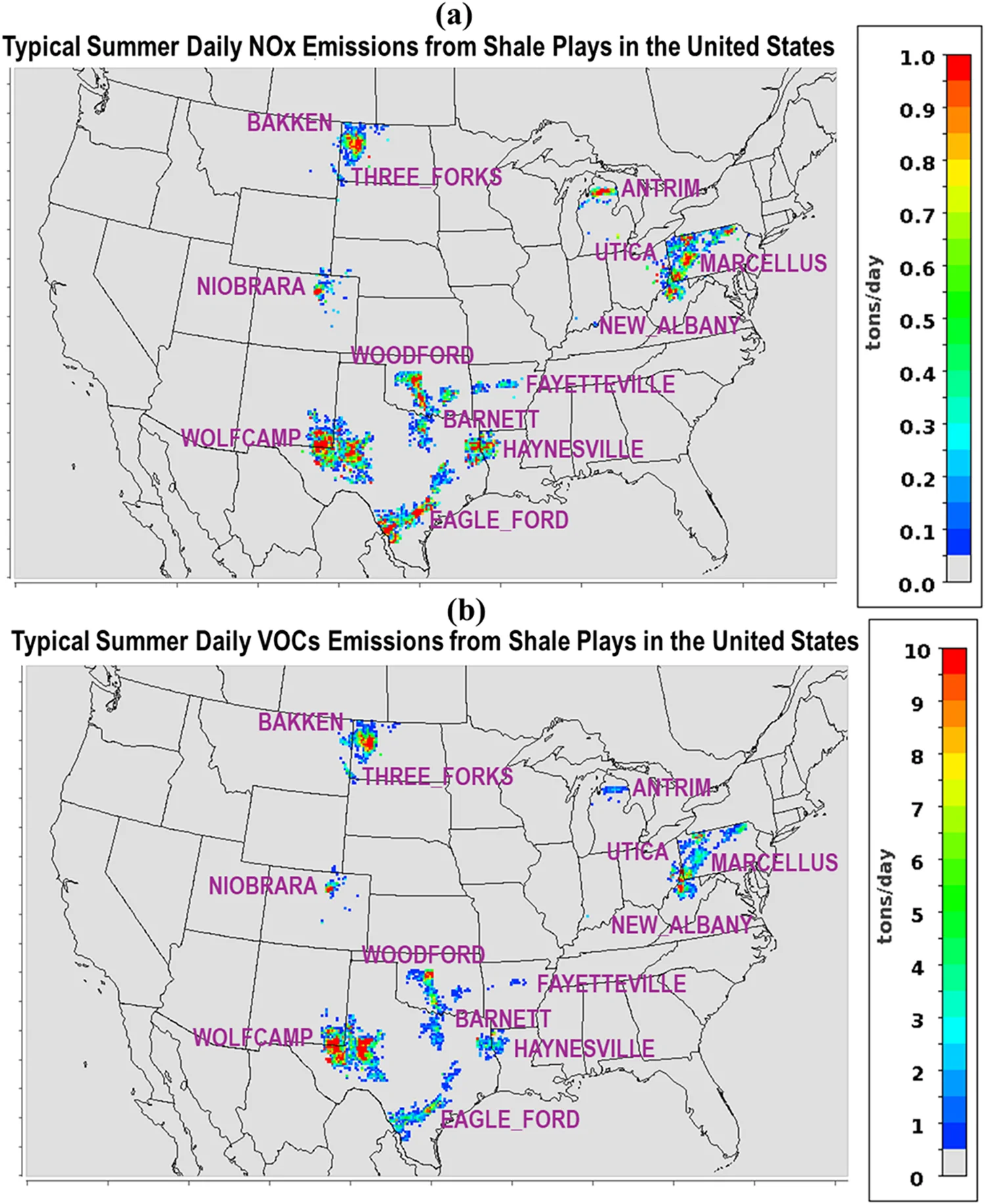
Typical daily NO_*x*_ (a) and VOC (b) emissions (in units of short tons/day/grid) from major shale plays (labeled in purple) in the United States.

### 2.2 Source Apportionment Modeling

Common approaches used to conduct CTM-based source-impact assessments include OSAT/PSAT, ISAM, brute-force, and the Decoupled, Direct Method, or DDM-3D. The brute-force method involves executing a model (at least) twice, once for a base case, and the second time where the emissions from a specific source are perturbed (e.g., reduced by a specified amount). DDM-3D involves simultaneously solving a second set of equations for the sensitivities of the concentrations to emissions and then using a Taylor series approach to calculate the source impact. Here, we chose to use ISAM to track how individual groups of sources impact O_3_ and air toxics.^76,77^ To use ISAM, the user specifies emissions (i.e., tags) for internal tracking by the model. This specification can pertain to emitted species (e.g., NO_*x*_ or VOCs or both), emission categories (e.g., emission from UOGD sources or other anthropogenic sources), or geographic designations (e.g., emissions from a basin). ISAM computes contributions to concentrations of related model species as source-apportioned values (i.e., percentage of the total concentration) for each tag. Depending on the computational load and resources (i.e., given the size of the domain and the chemical mechanism used), ISAM can accommodate up to a dozen source categories in one simulation. Considering these constraints, we configured the source apportionment for the following source groupings/categories (tags): UOGD emissions (subsectors of “np_oilgas” and “pt_oilgas”) from shale plays of Antrim and New Albany (tag name ANA), shale plays of Barnett, Fayetteville and Woodford (BFW), shale plays of Bakken and Three Forks (BTF), shale plays of Eagle Ford and Wolfcamp (EFW), shale play Haynesville (HAY), shale plays of Marcellus and Utica (MAU), shale play of Niobrara (NIO), non-UOGD oil and gas emissions (NUO), all other surface and elevated anthropogenic emissions (ANT) and biogenic emissions (BIO). In addition to the above, ISAM also tracks contributions from the initial (ICO) and boundary (BCO) conditions, and the “others” (OTH).

ISAM was set to track the OZONE class, which includes O_3_, all NITRATE species in the CB6r5 mechanism and aero7 module (i.e., ANO_3_, HNO_3_, NO, NO_2_, NO_3_, HONO, N_2_O_5_, PNA, PAN, PANX, NTR1, NTR2, INTR, CLNO_2_, CLNO_3_), and all VOC species (all the VOC species of the CB6r5 mechanism plus CO). Moreover, ISAM is configured to toggle between two weighting sets based on VOC- and NO_*x*_-limited ozone production in the reaction for ozone-NO_*x*_ chemistry, where the transition ratio of production of H_2_O_2_ over production of HNO_3_ is set as 0.35. When the ratio is less than 0.35, the weights for the case of VOC-limited ozone production are used; otherwise, the weights for the case of NOx-limited ozone production are used. More details are provided in Kwok et al.^76,77^

### 2.3 Observations and Model Evaluation

Model evaluation for simulated fields of O_3_ and other species followed the guidelines of Emery et al.^105^ and included various measures of bias and error recommended by the USEPA.^108^ The comparisons are performed by matching observations at each monitoring site to simulations in the modeling cell enclosing the site (i.e., matched in space) and focusing on the normalized mean bias and error and correlation coefficient (eqs S4–S6). This ensures that the apportioned concentrations are realistic and correspond to those measured. Special campaign data were used in the evaluation, such as the benzene measurements. Measurements of ozone, air toxics (e.g., formaldehyde and benzene), and particulate matter were collected during June 2023-December 2024 by a set of intensive measurement campaigns being conducted in three oil-producing states (NM, TX, CO) as part of the HEI-Energy TRACER campaigns.^56,109,110^ Satellite retrievals were also used for qualitative model evaluation. Several satellites have instruments that can observe trace pollutants, including nitrogen dioxide (NO_2_), one of the pollutants of concern coming from UOGD operations, providing vertical column densities (VCDs) of the molecules, as opposed to concentrations.^111^ VCDs are a measure of total columnar mass, which has proven useful for conducting trend analysis of pollutant emissions,^112^ and can be used to assess how well emissions are estimated, spatially, by comparison with air quality model calculations of VCDs.^113,114^ If the photochemical model results and observed VCDs are similar, it supports that emissions of the related species are well estimated and are particularly useful for spatial comparisons. Here, we use observations of NO_2_ and HCHO VCDs from TROPOMI^115–117^ and TEMPO^118^ for comparison to results from CMAQ. While most of the emissions of NO_*x*_ are in the form of nitric oxide (NO), in the presence of ozone or the peroxy radicals, NO reacts to form NO_2_, which is the more common form of NO_*x*_ during the day, except in very high NO_*x*_ areas near the source, such that NO_2_ can be used to identify sources of NO_*x*_.^119^

## 3 Results

### 3.1 Air Quality Model Performance Evaluation against Surface Monitors

We evaluated the base air quality modeling performance against surface measurements from the Air Quality System (AQS) national monitoring networks to ensure confidence in concentration predictions. For the CONUS domain, the simulations of Maximum Daily 1-h Average (MDA1) O_3_ and Maximum Daily 8-h Average (MDA8) O_3_ both met performance criteria (−15% < MNB < +15%, MNE < 25%, and *R* > 0.50) specified by Emery et al.^105^ for both 2023 and 2024 (Table 1, Figure S1). Other ozone-related gaseous species, including 24-h benzene, also achieved good performance with a relatively small NMB and NME and significant r; among them, 1-h NO_2_, 1-h NO_*y*_, and 24-h isoprene all having *r* > 0.5 for both years (Table 1, Figure S1) (see supplement for additional analyses, including seasonal evaluations, particularly Tables S4–S8). We also evaluated CMAQ simulations against benzene measurements from the special campaign averaged at a length of 24-h and week-long intervals, respectively. Results show that CMAQ captured weekly averaged benzene variations better than the shorter term (24-h), with an *R* ^2^ of 0.70 versus 0.49 at the Loving, NM site (Figure 3) and an *R* ^2^ of 0.55 versus 0.27 at the sites near Boulder, CO (Figure S2), likely due to both the intermittent nature of UOGD emissions^50,55^ and the coarseness of the grid. Peak levels of benzene can be tied to more local activities below the grid resolution of the CMAQ.

**Figure 3:**
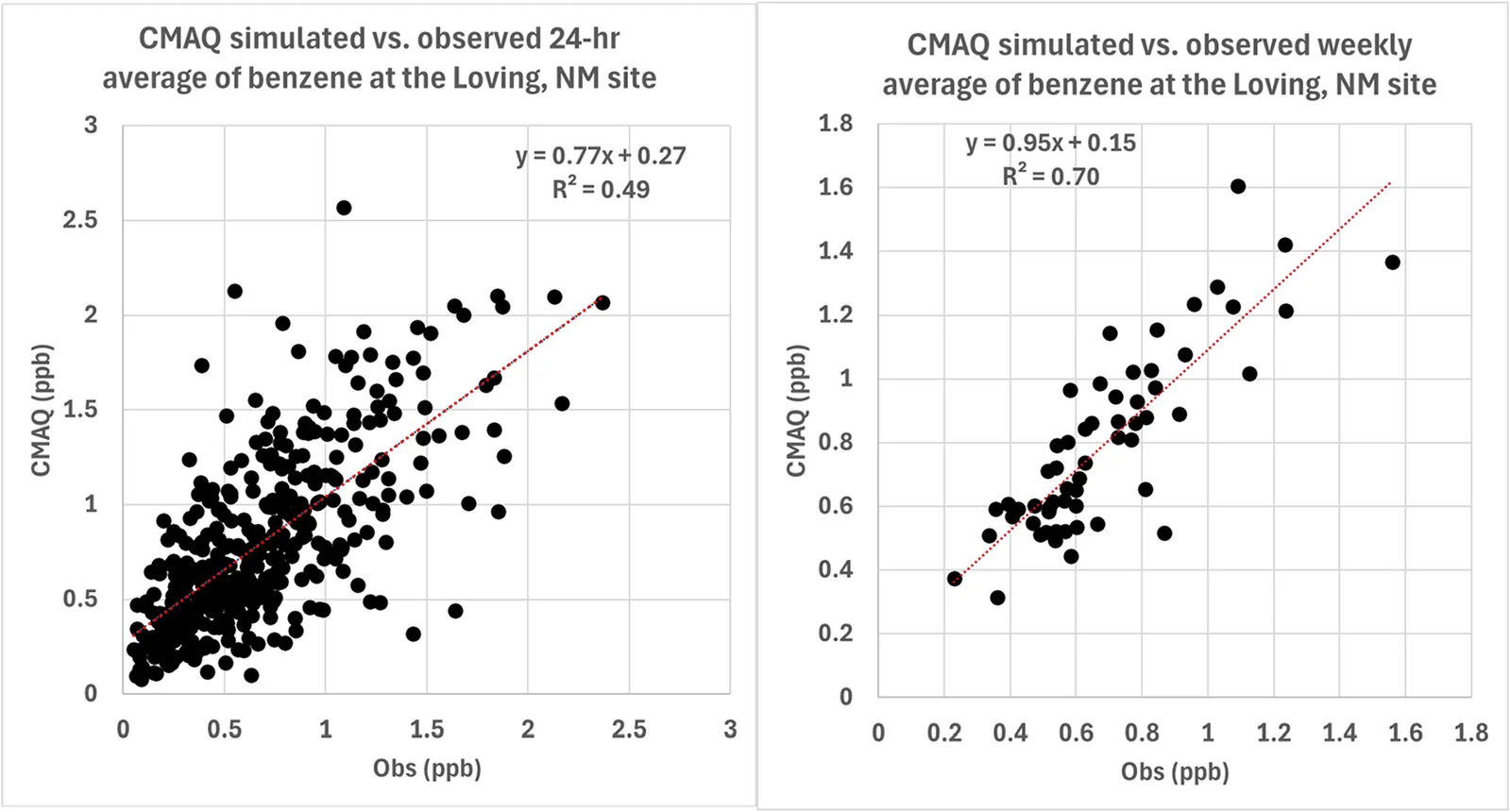
Scatter plots of CMAQ simulated 24-h benzene (left) and weekly benzene concentrations (right) against the averaged observations at the Loving, NM site (LNM).

**Table 1:** Annual Model Performance for Ozone and Its Related Species (no cutoff used)

Period	Variable	Unit	#Sites	#Pairs	Obs^a^	Sim^a^	NMB (%)	NME (%)	*r*
2023	MDA1 O_3_	ppb	1221	376395	47.39	41.55	–12.32	20.87	0.68
	MDA8 O_3_	ppb	1221	370210	43.46	39.01	–10.25	19.79	0.68
	24-h benzene	ppb	179	14573	0.19	0.11	–41.37	99.81	0.32
	24-h isoprene	ppb	54	6323	0.3	0.21	–29.25	95	0.67
	24-h toluene	ppb	173	14372	0.35	0.28	–20.05	74.13	0.26
	24-h formaldehyde	ppb	114	6574	2.84	1.07	–62.23	177.6	0.22
	1-h O_3_	ppb	1221	8982093	32.35	30.91	–4.43	28.19	0.70
	1-h NO_*y*_	ppb	83	614932	8.94	6.92	–22.55	77.1	0.55
	1-h NO_2_	ppb	444	3582426	7.64	4.86	–36.41	92.05	0.59
2024	MDA1 O_3_	ppb	1235	374707	47.03	41.43	–11.91	20.05	0.72
	MDA8 O_3_	ppb	1235	368403	43.12	38.82	–9.98	18.98	0.72
	24-h benzene	ppb	161	15309	0.18	0.11	–37.01	90.62	0.20
	24-h isoprene	ppb	54	7995	0.29	0.22	–23.85	93.62	0.63
	24-h toluene	ppb	158	14883	0.35	0.3	–15.93	70.92	0.16
	24-h formaldehyde	ppb	117	6398	2.46	1.09	–55.63	133.5	0.55
	1-h O_3_	ppb	1235	8934125	32.19	30.71	–4.6	27.61	0.71
	1-h NO_*y*_	ppb	84	612984	8.59	7.01	–18.36	73.22	0.56
	1-h NO_2_	ppb	459	3533628	7.49	4.95	–33.91	88.98	0.59

a Annual averages.

### 3.2 Comparison of Simulated NO_2_ and HCHO VCDs to Satellite Observations

Routine monitoring networks measure air pollutant ground-level concentrations at sparse locations. Furthermore, the monitors are usually located at more populated spots. Therefore, they are not necessarily capturing the influence of UOGD activities. Comparing the simulated hour 13–14 LST (time of the satellite overpass) averages of NO_2_ and HCHO VCD distributions with satellite-based instrument retrievals can further support that the spatial distribution of emissions is captured, including for UOGD emissions from major shale plays. Simulated NO_2_ VCDs were compared against both the TROPOMI and TEMPO retrievals for the period of August 1, 2023 through July 31, 2024 (Figure 4a and b). Quantitative agreement for each map was evaluated using spatial statistics computed from grid-cell–paired values, including the Pearson correlation coefficient (r), and normalized mean bias (NMB) (see Figure S3).

**Figure 4:**
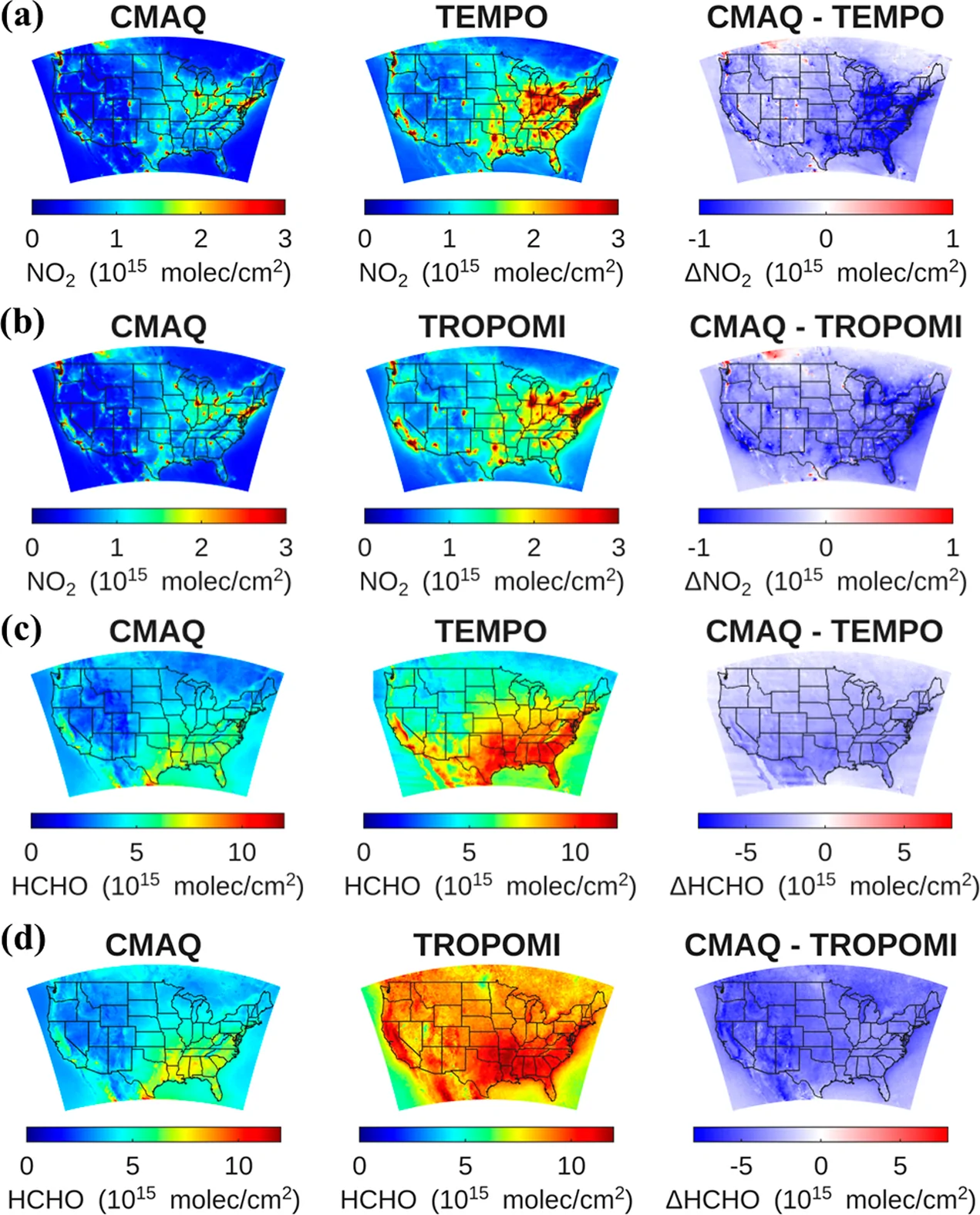
CMAQ simulated NO_2_ and HCHO VCDs compared against the TEMPO and TROPOMI retrievals for the period of August 1, 2023 through July 31, 2024: (a) TEMPO NO_2_, (b) TROPOMI NO_2_, (c) TEMPO HCHO, (d)­TROPOMI HCHO. TEMPO L2 v2 and TROPOMI L2 v2 retrievals (retrieved through the Remote Sensing Information Gateway) at 1:30 Local Solar Time were used and further screened by cloud with CF ≤ 0.15 and regridded to the 12US2 grid. The CMAQ fields were masked for availability of the corresponding satellite retrievals.

In general, CMAQ, TROPOMI, and TEMPO NO_2_ VCDs have similar spatial patterns, as reflected by high spatial correlations for each individual comparison (*r* = 0.88 and 0.90 for TROPOMI NO_2_ and TEMPO NO_2_, respectively). Some discrepancies were found. CMAQ is somewhat lower than the satellite VCDs over much of the domain (NMB = −37 and −38% for TROPOMI and TEMPO, respectively), particularly in areas with very low VCDs observed, and part of this is likely due to lower upper-tropospheric NO_2_ resulting from a low bias in upper-level NO_*x*_ emissions (lightning, aircraft).^120^ The bias grows moving eastward, which can be associated with processes leading to shorter modeled NO_2_ lifetimes or bias in upper-level emissions. Areas where CMAQ is locally biased high indicate emission estimates are biased high locally: for example, a portion of the Bakken in North Dakota, some cities (Seattle, Portland, and Salt Lake City), and the Mexico-US border area. These localized regions exhibit elevated NMB, consistent with systematic overestimation in specific emission regions as has been documented in other studies.^121^

HCHO VCDs show larger discrepancies than the NO_2_ VCDs between CMAQ versus TEMPO and TROPOMI retrievals (CMAQ NMB is −50.8% for TROPOMI, −38.8% for TEMPO), and between TEMPO and TROPOMI (TEMPO is 20–30% lower than TROPOMI across the domain) as well (Figure 4c and d). Spatial agreement is weaker than for NO_2_, with lower correlations for each comparison (*r* = 0.84 for CMAQ vs TEMPO and *r* = 0.54 for CMAQ vs TROPOMI), consistent with the larger magnitude of bias. However, the general patterns of the observed spatial distributions of HCHO VCDs were still captured by CMAQ simulations. For example, the higher levels of HCHO found in the southern portion of the domain, especially in the southeastern US where much stronger photochemistry produced more secondary HCHO with abundant biogenic VOC emissions, and the relatively higher levels over other areas reflecting HCHO and VOC emission distributions across the domain, for instance, the Central Valley in California, including the UOGD emissions from major shale plays, for instance, a portion over the Wolfcamp at the southeast corner of New Mexico. The differences, while significant, are consistent with prior work, which highlights a need for detailed evaluation of emissions inventories related to UOGD.^122^

### 3.3 Simulated Spatial Distributions of Ozone, Benzene, HCHO, and NO_2_

Before focusing on the source-impact results found using ISAM, spatial distributions of ozone and benzene and ground-level HCHO and NO_2_ are also investigated for perspective. Annual maximums of MDA8 O_3_ and 1-h averages of O_3_ are highest over and downwind of metropolitan areas (e.g., Los Angeles, New York City, Chicago, etc.), the Great Lakes, the East Coast, and over major shale plays such as the Wolfcamp (aka Permian: at the southeast corner of New Mexico extending into Texas), Denver-Juleberg Basin of Colorado, Eagle Ford (Texas), Bakken (North Dakota) and Woodford (Texas), Uinta Basin (Utah) (Figure 5a).

**Figure 5:**
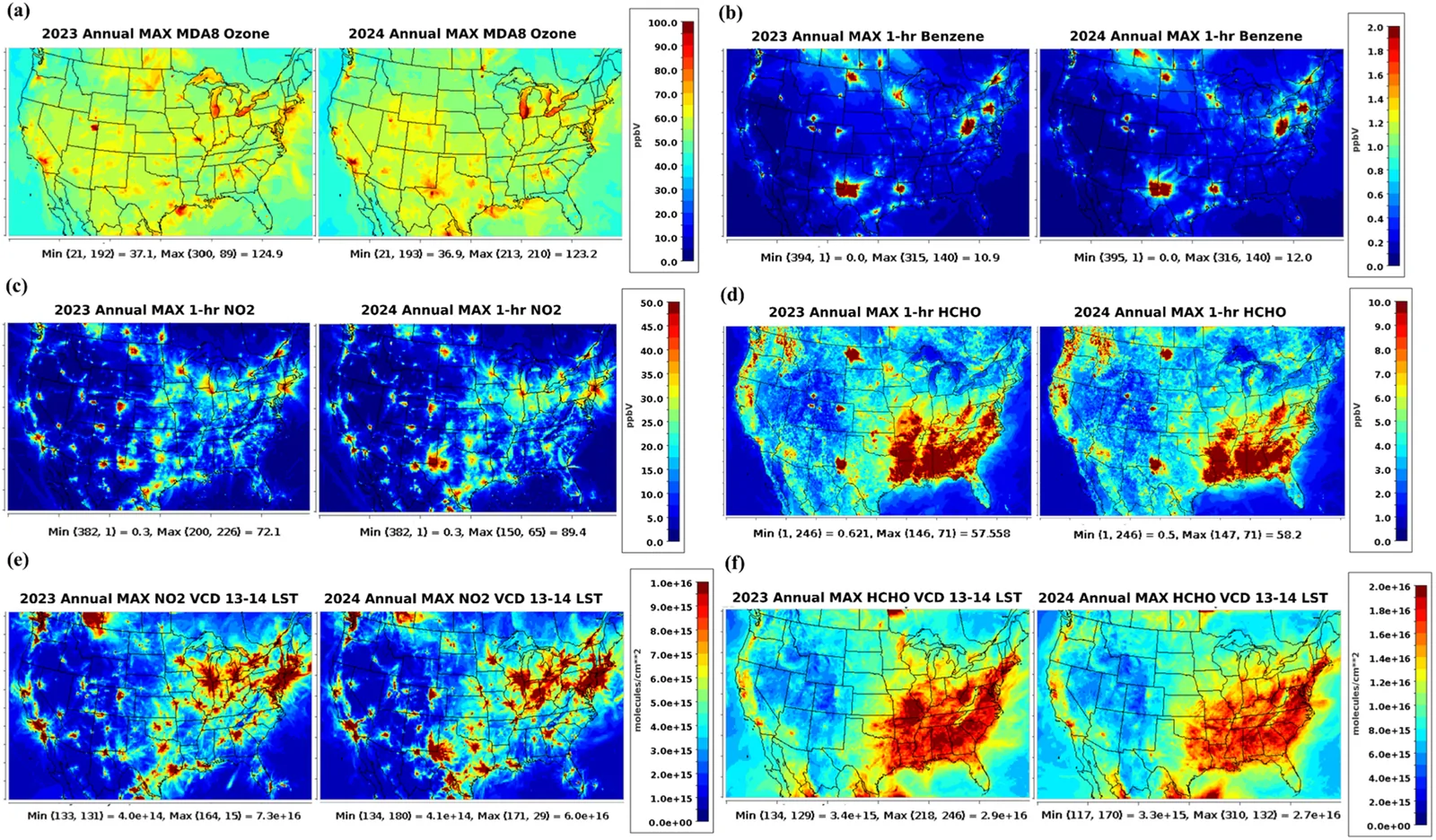
CMAQ simulated maximum surface MDA8 O_3_ (a), 1-h average of benzene (b), NO_2_ (c), and formaldehyde (HCHO) (d) concentrations, and 13–14 LST averages of NO_2_ VCD (e) and formaldehyde (HCHO) VCD (f) in 2023 (left column) and 2024 (right column).

Ground-level benzene, NO_2_, and HCHO also have elevated levels in urban areas and over major UOGD areas (Figure 5b,c,d), though HCHO, similar to the VCDs, is elevated over much of the Southeast, as HCHO is a product of reactions of the plentiful biogenic VOC emissions in that area. Primary species such as benzene are geographically more aligned with the major shale plays, while NO_2_ stands out over both shale plays and metropolitan areas, somewhat like ozone (Figure 5).

### 3.4 Source Apportionment Results for Major Shale Plays

Air quality impacts for each of the 7 tagged shale plays (ANA, BFW, BTF, EFW, HAY, MAU, NIO) were calculated individually using ISAM (though they are merged in Figure 6 for compactness). UOGD activities lead to elevated O_3_ and NO_2_ nationally (Figure 6a,b). UOGD VOCs and NO_*x*_ emissions lead to increased ozone formation, both more locally and continentally, with maximum MDA8 O_3_ levels greater than 15 ppb over much of the eastern US and elevated levels are found in California due to transport. UOGD emissions are also responsible for NO_2_ impacts >10 ppb over large areas, including near the shale play locations and spreading out metropolitan areas such as Denver, CO. Compared to UOGD impacts on surface NO_2_, UOGD impacts on NO_2_ VCDs over many urban areas are more substantial, e.g., in Los Angeles and New York City (Figure 6c). This indicates that UOGD NO_*x*_ emissions and UOGD-related ozone are transported over long distances.

**Figure 6:**
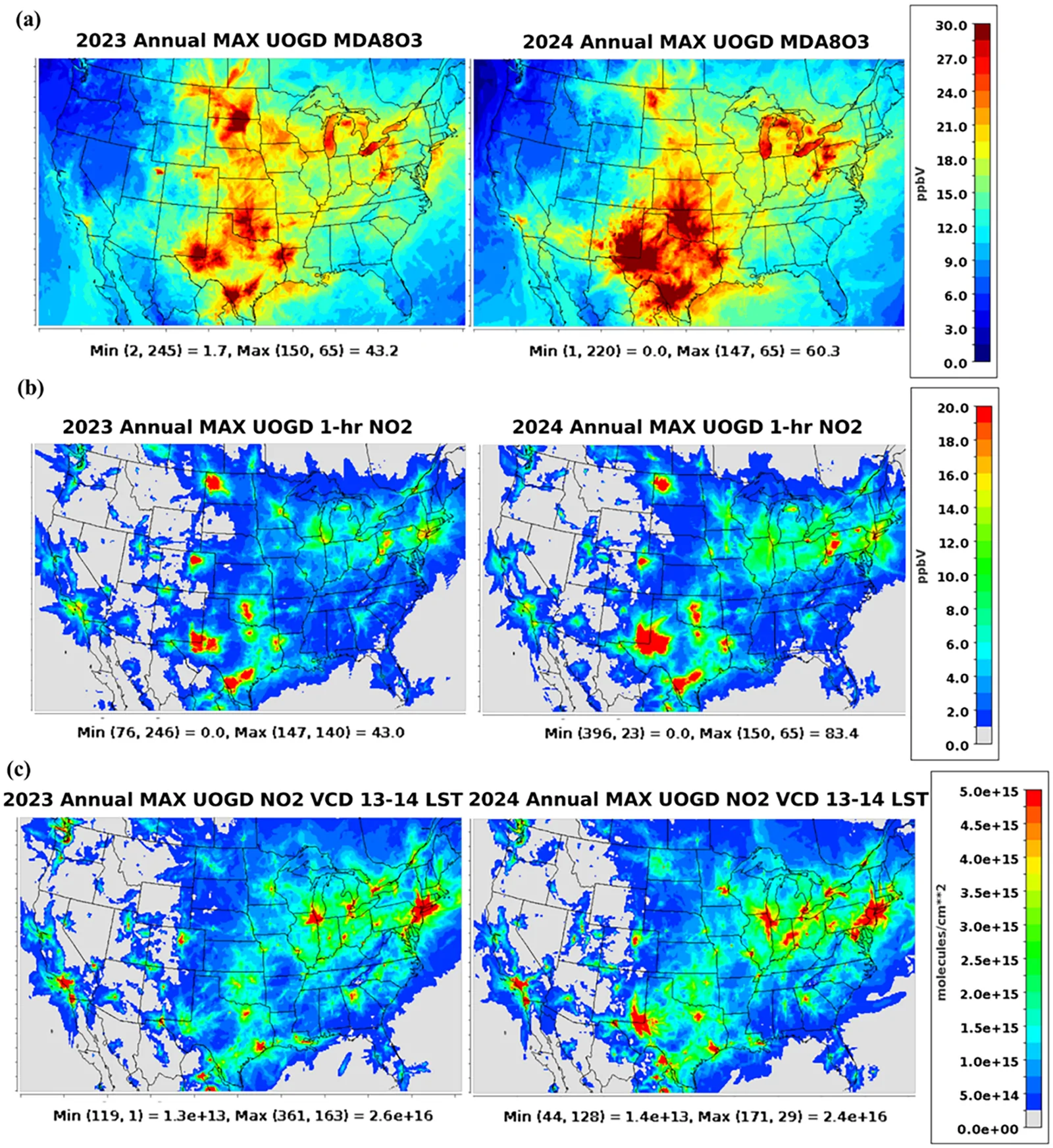
CMAQ-ISAM modeled maximum UOGD contribution to MDA8 O_3_ concentrations (a), surface hourly NO_2_ (b), and 13–14 LST mean NO_2_ VCD (c) in 2023 (left column) and 2024 (right column).

At locations near a major shale play, UOGD contributions to MDA8 O_3_ can be high (Figure 7 and Figure S4). For example, at the special campaign monitoring site in Loving, New Mexico (which has nonattainment levels of ozone), the Eagle Ford-Wolfcamp (EFW) shale play contributes much higher than other anthropogenic sectors (i.e., the ANT and NUO) at most of the time throughout the year, and can contribute over 25 ppb to MDA8 O_3_ during summer, while more distant shale plays can also contribute 1∼2 ppb, especially in cooler seasons (Figure 7). The single-site analyses presented here are intended to provide illustrative examples of UOGD impacts at locations influenced by intensive oil and gas activities and for which field campaign observations were available. These sites were selected to evaluate model performance and source contributions under conditions representative of major UOGD regions. However, because the analyses are based on observations collected during relatively limited periods, the results may not fully represent long-term conditions or the full range of meteorological variability. Therefore, the single-site results should be interpreted in conjunction with the broader regional-scale source-attribution results, which provide a more robust assessment of the average impacts of UOGD emissions on air quality.

**Figure 7:**
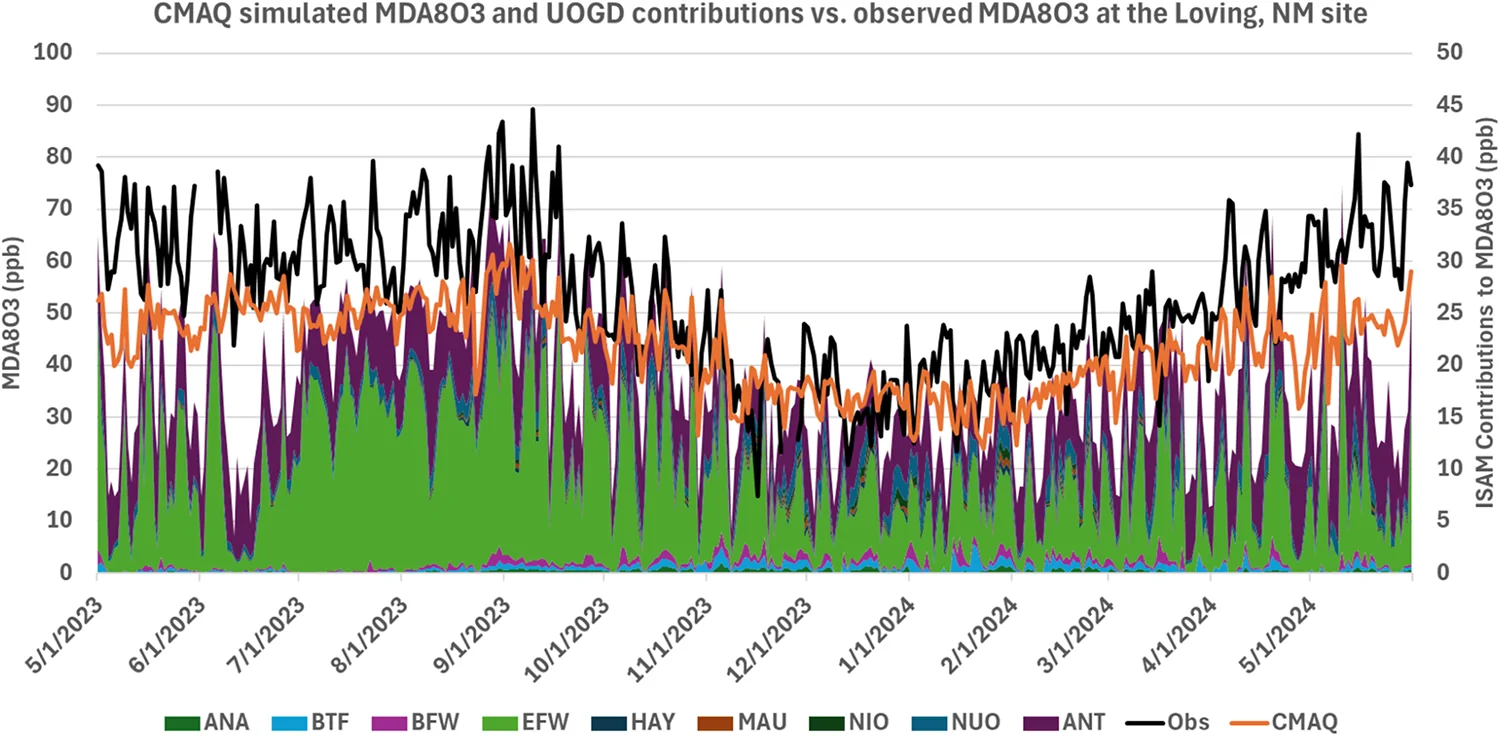
CMAQ-ISAM simulated time series of major shale plays’ contributions to MDA8 O_3_, with simulated MDA8 O_3_ concentration against the observed at the Loving, New Mexico site (LNM). LNM site is located within the Wolfcamp shale play, specifically in the Delaware Basin, which is the western sub-basin of the larger Permian Basin. Also shown are contributions from other anthropogenic sectors of NUO (non-UOGD oil and gas) and ANT (other anthropogenic, including on-road emissions). The largest contributor is “background,” which is not shown to make the other contributions more evident.

To provide a broader regional perspective, we also analyzed UOGD contributions to MDA8 O_3_ at three additional AQS sites: Rubidoux, California; Camp Bullis, Texas; and South Fayette, Pennsylvania (Figures S5–S7). As expected, the Marcellus–Utica (MAU) shale plays were the largest UOGD contributors at South Fayette, while the Eagle Ford–Wolfcamp (EFW) shale plays dominated at Camp Bullis. At Rubidoux, non-UOGD oil and gas (NUO) emissions contributed more to ozone than all UOGD shale plays combined; however, contributions from distant shale plays, particularly EFW, were evident during some seasons. Nevertheless, other anthropogenic sources (ANT) remained the dominant contributor to ozone at all three sites.

Compared to the maximum UOGD contributions to MDA8 O_3_ (Figure 6), the averaged UOGD contributions to MDA8 O_3_ are lower (Figure 8a,b). The maximum of the annual average contributions can be as large as over 10 ppb, with higher contributions during the ozone season (May-September), which can be as large as over 15 ppb, with lower contributions in colder months (Figure 8a,b).

**Figure 8:**
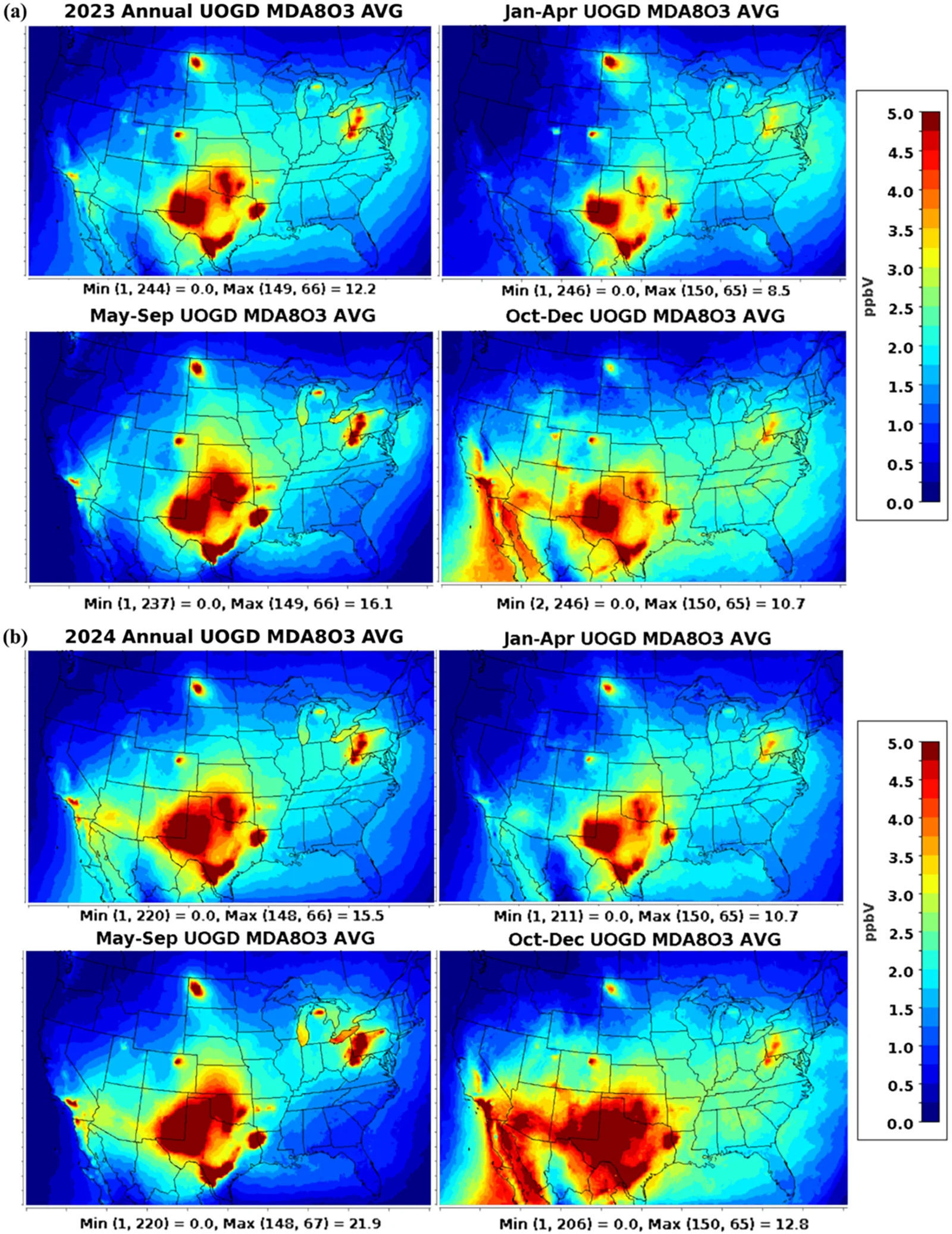
(a) 2023 annual and seasonal average of UOGD contributions to MDA8 O_3_ simulated by CMAQ-ISAM: annual (top left), Jan-Apr (top right), May-Sept (bottom left), and Oct-Dec (bottom right); (b) 2024 annual and seasonal average of UOGD contributions to MDA8 O_3_ simulated by CMAQ-ISAM: annual (top left), Jan-Apr (top right), May-Sept (bottom left), and Oct-Dec (bottom right).

To provide context for the reported maximum and average contributions, Figures S8 and S9 show the frequency distributions of daily UOGD contributions to MDA8 O_3_ in 2023 and 2024. Contributions exceeding 5 ppb occur in less than approximately 10% of grid cell/day combinations, indicating that the highest impacts are episodic rather than typical. On ozone exceedance days (MDA8 O_3_ > 70 ppb), however, UOGD contributions are substantially larger, averaging 6.26 ppb in 2023 and 9.27 ppb in 2024, compared with corresponding average MDA8 O_3_ concentrations of 75.2 and 75.5 ppb, respectively. These results indicate that UOGD emissions can play an important role during high-ozone episodes, even though their domain-wide average contribution is smaller.

The magnitude of the ozone impacts estimated here is generally consistent with that of previous studies. Prior investigations reported UOGD-related ozone enhancements of approximately 3 ppb on seasonal-average summer days,^51^ 20 ppb at downwind national park locations,^57^ and more than 30 ppb during individual high-ozone episodes.^54^ These published values support the plausibility of the ozone contributions estimated in this study and demonstrate that UOGD emissions can substantially affect air quality from local to regional scales and over long distances.

Note that, when using ISAM, the UOGD impact on Los Angeles’ MDA8 O_3_ can be 3–4 ppb in 2023 and 4–5 ppb in 2024 on average during the ozone season (Figure 8a,b), and larger in both 2023 and 2024 during October-December under specific meteorological regimes that favored westward transport into Southern California (e.g., trajectory-based analysis shown in Figure S10). Also note that, in a large area of the Southern US and Northern Mexico, the UOGD impacts on MDA8 O_3_ during the October-December period are also elevated and higher than the impacts during other months (compare Figure 8a and b).

Given the importance of the downwind impacts found (e.g., in nonattainment areas of California), a brute-force simulation with emissions from the shale plays of Eagle Ford and Wolfcamp being reduced by 30% was conducted for periods with large impacts in California. The brute-force sensitivity was then calculated by renormalizing the concentration difference between the base case and the emissions reduction case to the level as if 100% emissions were absent. A spatially similar, but smaller, downwind impact was found (∼1 ppb compared with ∼4 ppb in the ISAM results; Figure S11a). Given the differences regarding the quantitative magnitude of the contribution, additional investigation of the strengths and limitations of different source apportionment techniques is needed. In particular, ozone-tagging approaches may overestimate source contributions because they do not fully account for ozone reductions associated with NO_*x*_-related titration and nonlinear chemical interactions.^78^ The impacts on more primary species, including NO2 and benzene, were not impacted so much (Figure S11b,c).

The averaged UOGD contributions to surface NO_2_ are similar to MDA8 O_3_ spatially but more concentrated in shale plays and their surrounding areas (Figure S11a,b). UOGD contributions to NO_2_ VCDs do impact far downwind populated areas, e.g., Los Angeles and New York City, particularly during the colder months; the impacts in these metropolitans are much more outstanding, even higher than in the shale play areas (Figure S12a,b).

UOGD emissions lead to elevated benzene (over 1 ppb) and HCHO (over 5 ppb) for both years (Figure S13). While the benzene increases are spatially limited, HCHO impacts have a slightly increased footprint due to secondary formation, although the maximum impacts are in the shale plays themselves, e.g., from direct emissions by flares.

UOGD contributions to benzene can be significant compared with other anthropogenic contributions at specific urban/suburban locations near a major shale play. For example, at special campaign monitoring sites in Broomfield, CO (near both Boulder and Denver), the Niobrara (NIO) shale play consistently contributes 30–50% of the simulated weekly benzene concentrations, which is comparable to all other anthropogenic sources’ contributions together (Figure S14), while at the Loving NM site (LNM), the Eagle Ford-Wolfcamp (EFW) shale play dominates the benzene contributions throughout the year (Figure 9). Previous studies also identified benzene as an important UOGD-related air toxic,^26,52^ while our analysis extends these findings by quantifying source-specific benzene contributions at continental scale.

**Figure 9:**
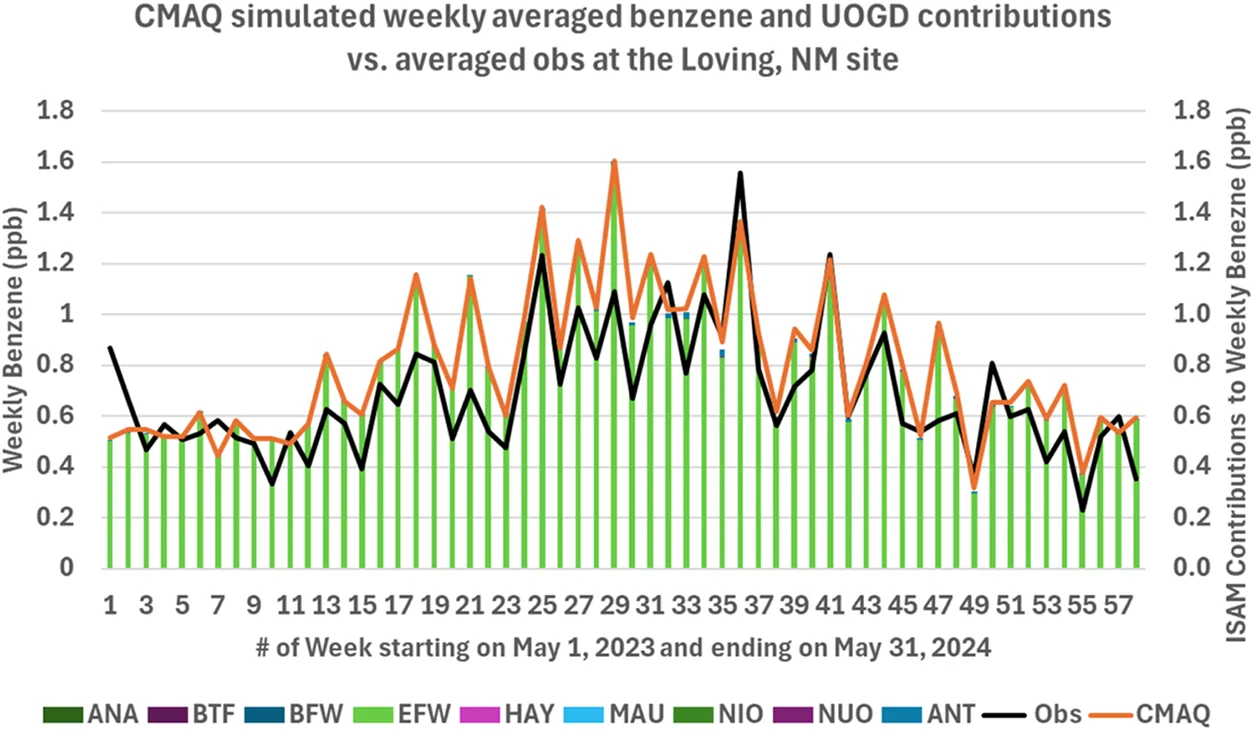
CMAQ simulated time series of major shale plays’ contributions to weekly averaged benzene concentration with simulated weekly benzene concentration against observations at the Loving, New Mexico site (LNM). The Eagle Ford–Wolfcamp (EFW) shale plays dominate the benzene contributions throughout the year. Contributions from other UOGD shale plays and non-UOGD oil and gas sources (NUO) are negligible, while other anthropogenic sources (ANT) contribute only occasionally and at relatively low levels.

### 3.5 Population-Weighted Contribution to Exposures from Major Shale Plays

We calculated the US population-weighted annual averages and annual maximums of MDA8 O_3_, 1-h benzene, 1-h NO_2_, and 1-h formaldehyde (HCHO), and UOGD contributions to them as simulated by CMAQ-ISAM within the CONUS domain using the 2023 population data (https://data.census.gov). Annual average UOGD contributions to population-weighted MDA8 O_3_ found using ISAM were 0.9 ppb in 2023 and 1.0 ppb in 2024, respectively, about double the non-UOGD oil and gas contributions, and two-thirds of the biogenic contribution but one-quarter of other anthropogenic contributions (Figure 10a). ISAM calculated annual maximum UOGD contributions to MDA8 O_3_ were 11 ppb in 2023 and 12 ppb in 2024, slightly exceeding the biogenic contributions and about more than half of other anthropogenic contributions (Figure 10b). This indicates that UOGD emission impacts can travel long distances and impact densely populated areas. However, as found above, the magnitude of the ozone impacts can differ based on the methods used.

**Figure 10:**
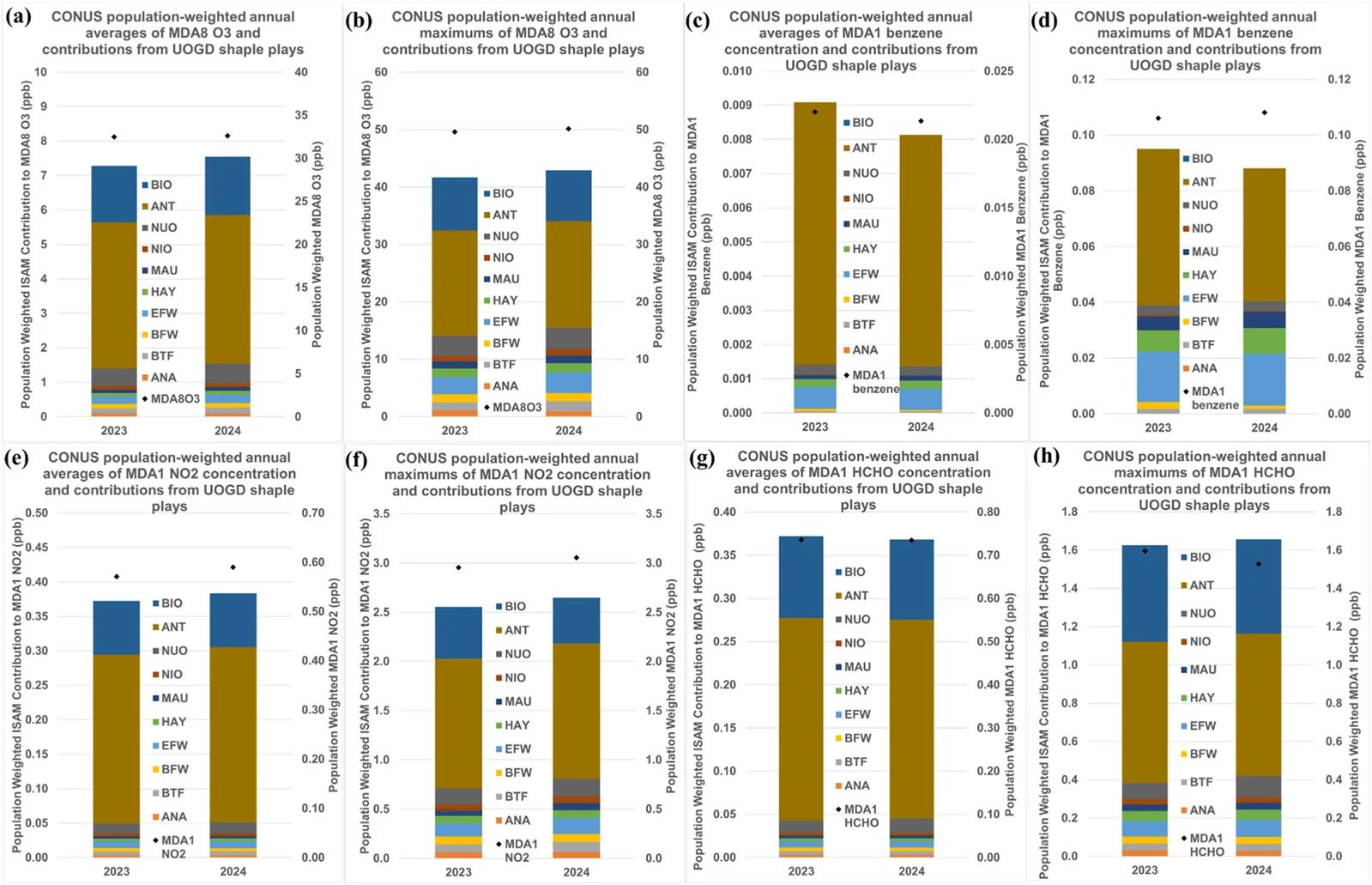
CONUS population-weighted annual averages (left column) and annual maximums (right column) of MDA8 O_3_ (panels (a) and (b)), MDA1 benzene (panels (c) and (d)), MDA1 NO_2_ (panels (e) and (f)), and MDA1 formaldehyde (HCHO, panels (g) and (h)), and their UOGD shale plays’ and other sectors’ contributions simulated by CMAQ-ISAM. The ISAM TAG names presented in the figure are defined as ANA, shale plays of Antrim and New Albany; BFW, shale plays of Barnett, Fayetteville and Woodford; BTF, shale plays of Bakken and Three Forks (BTF); EFW, shale plays of Eagle Ford and Wolfcamp; HAY, shale play Haynesville; MAU, shale plays of Marcellus and Utica; NIO, shale play of Niobrara (NIO); NUO, non-UOGD oil and gas emissions; ANT - all other surface and elevated anthropogenic emissions; and BIO, biogenic emissions. Note that the black diamonds display the concentrations (refer to the right *y*-axis) while the color bars display the contributions (refer to the left *y*-axis).

The annual average, population-weighted UOGD contributions to 1-h benzene are ∼1.1 ppt in both years, about 40% of the other anthropogenic contributions (e.g., from mobile sources, Figure 10c). The annual maximum UOGD contributions to 1-h benzene are 35 ppt in 2023 and 37 ppt in 2024, respectively, about 70% of other anthropogenic contributions (Figure 10d).

The annual average, population-weighted UOGD contributions to 1-h NO_2_ are ∼0.035 ppb in both years, about three times the non-UOGD oil and gas contributions, but only ∼15% of all other anthropogenic contributions (Figure 10e). The annual maximum UOGD contributions to 1-h NO_2_ are 0.55 ppb in 2023 and 0.63 ppb in 2024, respectively, slightly more than the biogenic contributions, and about 40% of other anthropogenic contributions (Figure 10f).

The annual average population-weighted UOGD contributions to 1-h formaldehyde are ∼0.03 ppb in both years (Figure 10g). Annual maximum UOGD contributions to 1-h formaldehyde are ∼0.3 ppb in both years, about two-thirds of the biogenic contributions, and 40% of other anthropogenic contributions (Figure 10h).

## 4 Discussion

Unconventional oil and gas development (UOGD) has been increasing over the last three decades and now accounts for most of the oil and natural gas produced in the US. The related UOGD emissions include ozone and particulate matter precursors as well as air toxics such as benzene. Regional impacts of UOGD on ozone and air toxics were assessed by using CMAQ-ISAM to directly link UOGD activity to ambient air quality. ISAM proved to be an efficient approach for estimating impacts of UOGD emissions from different production basins. However, it also has a limitation, as the formulation of ISAM involves a heuristic that allocates the emissions-air quality impacts on ozone to either NO_*x*_ or VOC emissions, not their combination, and is mathematically inexact. Further, it does not account for negative impacts of NO_*x*_ on ozone, such as titration, which can bias the results. Our results suggest a bias in the downwind impacts of ozone found by using ISAM. Additionally, potential inaccuracies in the magnitude and chemical composition of UOGD emissions also play an important role in source-attribution analyses. In particular, the speciation of VOC emissions can vary substantially among shale basins, production stages, and inventory development approaches.^55,56,110^ Because ozone formation and air toxic concentrations are sensitive to both the quantity and composition of emitted VOCs, uncertainties in VOC speciation may influence the absolute magnitude of the simulated ozone and benzene impacts reported here. While the use of EPA inventories provides a framework for evaluating UOGD impacts across the United States, future studies incorporating improved basin-specific emission characterizations, updated VOC speciation profiles, and additional field observations could further reduce these uncertainties and improve estimates of UOGD contributions to ozone and air toxics.

ISAM results show that UOGD activities lead to elevated O_3_, NO_2_, benzene, and formaldehyde both locally and regionally. UOGD was found to contribute over 15 ppb to MDA8 O_3_ over large areas in the CONUS when using ISAM. UOGD emissions are responsible for NO_2_ maximums of >10 ppb over much of the US due to transport. Population-weighted contributions provide better estimates of the population’s actual exposure to UOGD-influenced air pollution. We found that UOGD contribute to US population-weighted annual averages of MDA8 O_3_, 1-h benzene, 1-h NO_2_, and 1-h formaldehyde (HCHO) concentrations at about 1 ppb (3%), 1.1 ppt (5%), 0.035 ppb (6%), and 0.03 ppb (4%), respectively. The annual maximum UOGD contributions increase by a large amount compared to their annual averages, both absolutely; for example, the maximum contributions to MDA8 O_3_, 1-h NO_2_, and 1-h formaldehyde are about 10 times the averages, while the maximum contributions to 1-h benzene are 35 times the average, and relatively compared to other emissions, for example, the annual maximum UOGD contributions to MDA8 O_3_ compared to the annual average increase from one-fourth to half of other anthropogenic contributions. Additionally, a comparison of CMAQ simulated NO_2_ and HCHO VCDs to satellite retrievals such as TEMPO and TROPOMI data reveals that, in general, CMAQ and satellite VCDs have similar spatial patterns.

An unexpectedly large impact (up to 4–5 ppb in ozone season) was found for UOGD emissions originating from shale plays further east on ozone in the Los Angeles area when using ISAM, though using a brute-force approach led to a lower impact. Southern California historically has some of the worst air pollution in the United States and continues to experience elevated levels of ozone. Despite significant controls that have led to improvements in air quality, this area continues to be in nonattainment with the National Ambient Air Quality Standards (NAAQS) for ozone, and the rate of decline in ozone levels has diminished, if not showing signs of reversal.^123^ The potential impact of such emissions is of importance, as is the difference in simulated impacts when the two different methods are used.

A primary contribution of this work is the quantification of local, regional, and long-range impacts of UOGD emissions on ozone and air toxics across the contiguous United States using a consistent source-attribution framework. The ability of CMAQ to simulate air toxics such as benzene and formaldehyde, as well as photochemically produced gases such as ozone and NO_2_, along with local-scale dispersion models,^55,56,110^ can support detailed exposure modeling at local to regional scale for health assessments of the impacts of UOGD operations on air quality and health. As found in this study, CMAQ did a better job of capturing longer-term (week-long, as tested here) variations of air toxic concentrations compared to shorter terms (including major peak levels), likely due to both the intermittent nature of UOGD emissions^55,56^ and the coarseness of the grid. As noted by Graves et al.,^55^ local-scale dispersion modeling was needed to capture peak levels, but emissions from up to 100 km away were needed to get the mean levels. Regional photochemical modeling provides secondary species impacts not currently captured by dispersion models, along with regional impacts of primary species, and provides a direct approach to linking regional exposures to source emissions for human health studies and regulatory analyses.

## Supplementary Material



## Notes

Meteorological and air quality model evaluation methods and results, including evaluation metrics, annual and monthly performance statistics for 2023 and 2024, and additional figures comparing model simulations with observations, modeled emissions from major shale plays and other source sectors across the contiguous United States, and supplementary figures illustrating UOGD contributions, transport pathways, and comparisons among modeling approaches (PDF)
