# Supplementary material for: Regional Impacts of Unconventional Oil and Gas Operations on Ozone and Air Toxics
Source: Environ Sci Technol. 2026 Oct 19;60(43):30737–53. doi: 10.1021/acs.est.6c03632 (PMC13738785; doi:10.1021/acs.est.6c03632)
Supplement: Supplementary file 1 [file es6c03632_si_003.pdf]

Supporting Information

Regional Impacts of Unconventional Oil and Gas Operations on Ozone and Air Toxics

Yongtao Hu[1], Kamal Jyoti Maji[1], M. Talat Odman[1], Jennifer Kaiser[1] and Armistead G. Russell[1]

[1]School of Civil and Environmental Engineering, Georgia Institute of Technology, Atlanta, GA, 30332, USA

Equations for calculating the bias error (Bias), Gross error (GE) and root mean square error (RMSE) for evaluation of meteorological parameters

$$Bias = \frac{1}{IJ}\sum_{j=1}^{J}\sum_{i=1}^{I}\left(P_j^i - O_j^i\right) \qquad \text{(S1)}$$

$$GE = \frac{1}{IJ}\sum_{j=1}^{J}\sum_{i=1}^{I}\left|P_j^i - O_j^i\right| \qquad \text{(S2)}$$

$$RMSE = \left[\frac{1}{IJ}\sum_{j=1}^{J}\sum_{i=1}^{I}\left(P_j^i - O_j^i\right)^2\right]^{1/2} \qquad \text{(S3)}$$

where N is the number of observation (O) and simulation (P) pairs for each meteorological variable.

Table S1 Annual surface meteorological model performance

Period	TMP Bias (Celsius)	TMP GE (Celsius)	RH Bias (%)	RH GE (%)	WS Bias (m/s)	WS RMSE (m/s)	WD Bias (deg)	WD GE (deg)
2023	-0.39	2.19	-6.74	12.6	-0.49	3.3	5.08	45.5
2024	-0.43	2.17	-6.1	12.0	-0.45	3.2	5.42	44.9

Table S2 Monthly surface meteorological model performance

Period	TMP Bias (Celsius)	TMP GE (Celsius)	RH Bias (%)	RH GE (%)	WS Bias (m/s)	WS RMSE (m/s)	WD Bias (deg)	WD GE (deg)
2023								
Jan	-1.24	2.46	-4.71	11.79	-0.44	3.57	4.83	44.91
Feb	-1.31	2.61	-1.45	12.63	-0.68	3.88	5.36	43.55
Mar	-1.31	2.37	-1.72	11.7	-0.69	3.72	5.75	42.63
Apr	-0.8	2.25	-1.52	11.74	-0.77	3.66	4.28	42.83
May	-0.24	2.03	-4.62	10.87	-0.56	3.18	4.97	45.63
Jun	0.19	2.12	-7.39	11.58	-0.46	3.03	4.71	48.19
Jul	0.48	2.31	-10.36	13.42	-0.44	2.99	4.02	50.27
Aug	0.38	2.19	-10.97	13.91	-0.43	2.97	3.97	47.84
Sep	0.03	1.96	-9.65	12.69	-0.41	2.89	5.53	47.61
Oct	-0.24	1.98	-10.11	13.24	-0.39	3.26	5.93	42.79
Nov	-0.4	2.04	-8.74	13.39	-0.33	3.21	5.63	43.54
Dec	-0.29	2.05	-8.95	14.28	-0.34	3.27	6.05	46.13
2024								
Jan	-1.21	2.49	-4.16	12.18	-0.35	3.2	6.08	45.17
Feb	-1.13	2.2	-2.39	12.14	-0.49	3.17	5.02	43.32
Mar	-0.83	2.09	-2.77	11.56	-0.61	3.28	4.78	42.38
Apr	-0.54	2	-3.54	11.18	-0.77	3.62	4.97	39.92
May	-0.21	2.11	-5.38	11.05	-0.54	3.42	5.53	45.57
Jun	-0.15	2.12	-6.35	10.78	-0.53	3.24	4.9	42.97
Jul	0.36	2.21	-8.18	11.74	-0.42	2.94	4.03	49.25
Aug	0.36	2.22	-9.54	12.75	-0.44	2.96	6.58	49.23
Sep	-0.22	2	-7.36	11.38	-0.26	2.86	6.16	45.66
Oct	-0.55	2.14	-8.05	12.05	-0.26	3.13	5.16	44.45
Nov	-0.56	2.08	-7.68	13.12	-0.39	3.35	6.1	43.34
Dec	-0.6	2.38	-7.52	13.97	-0.35	3.27	5.68	46.65

Table S3 Annual modeled NOx and VOCs emissions* from major shale plays and sectors in CONUS

Sum	TAG**	Shale play/Sector	NOx (short tons/yr)	VOCs (short tons/yr)
		ANTRIM	1.40E+04	1.16E+04
		NEW_ALBANY	2.66E+02	8.54E+02
	ANA		1.42E+04	1.24E+04
		BARNETT	1.05E+04	4.53E+04
		FAYETTEVILLE	6.45E+03	5.39E+03
		WOODFORD	4.45E+04	1.25E+05
	BFW		6.14E+04	1.75E+05
		BAKKEN	4.96E+04	2.55E+05
		THREE_FORKS	1.15E+03	4.83E+03
	BTF		5.07E+04	2.60E+05
		EAGLE_FORD	9.43E+04	1.69E+05
		WOLFCAMP	2.02E+05	1.05E+06
	EFW		2.96E+05	1.22E+06
	HAY	HAYNESVILLE	3.99E+04	6.41E+04
		MARCELLUS	7.11E+04	2.53E+05
		UTICA	7.35E+03	5.54E+04
	MAU		7.84E+04	3.09E+05
	NIO	NIOBRARA	1.63E+04	4.76E+04
UOGD total			*5.57E+05*	*2.09E+06*
	NUO	Non-UOGD Oil&Gas	4.74E+05	8.74E+05
Oil&Gas total			*1.03E+06*	*2.97E+06*
		Electric Generating	8.08E+05	2.80E+04
		Highway Vehicles	2.33E+06	1.04E+06
		Off-Highway	1.65E+06	1.07E+06
		Solvent Utilization	8.79E+02	2.74E+06
		Other	2.35E+06	8.49E+06
	ANT		7.14E+06	1.34E+07
anthropogenic total			*8.18E+06*	*1.63E+07*
	BIO	Natural Resources	9.81E+05	2.77E+07
CONUS total emissions			*9.16E+06*	*4.40E+07*

*Averaged for 2023 and 2024. Summed up to TAGs and other levels of total as well.

**TAGs were configured for the following source groupings/categories: UOGD emissions (sub-sectors of "np_oilgas" and "pt_oilgas") from shale plays of Antrim and New Albany (tag name ANA), shale plays of Barnett, Fayetteville and Woodford (BFW), shale plays of Bakken and Three Forks (BTF), shale plays of Eagle Ford and Wolfcamp (EFW), shale play Haynesville (HAY), shale plays of Marcellus and Utica (MAU), shale play of Niobrara (NIO), non-UOGD oil and gas emissions (NUO), all other surface and elevated anthropogenic emissions (ANT) and biogenic emissions (BIO).

Equations for calculating the normalized mean bias and error and correlation coefficient for air quality model performance evaluation

Normalized mean bias (NMB): $NMB = \frac{\sum_{i=1}^{N}(\mathrm{P_i}-\mathrm{O_i})}{\sum_{i=1}^{N}\mathrm{O_i}} X100$ (S4)

Normalized mean error (NME): $NME = \frac{\sum_{i=1}^{N}|\mathrm{P_i}-\mathrm{O_i}|}{\sum_{i=1}^{N}\mathrm{O_i}} X100$ (S5)

Correlation coefficient (r): $r = \frac{\sum_{i=1}^{N}[(\mathrm{P_i}-\overline{\mathrm{P}})X(\mathrm{O_i}-\overline{\mathrm{O}})]}{\sqrt{\sum_{i=1}^{N}(\mathrm{P_i}-\overline{\mathrm{P}})^2 X \sum_{i=1}^{N}(\mathrm{O_i}-\overline{\mathrm{O}})^2}}$ (S6)

where N is the number of observation (O) and simulation (P) pairs for each evaluated species, overbars signify the means.

Air quality model performance evaluation - additional analyses, including seasonal comparisons

Seasonally, model performance of MDA8 O_3 show a small negative biases with slight variations except April-June when NMB and/or NME exceeded the criteria (Table S5). This seasonal variation of performance could be caused by using the 2020/2021 emissions inventories for 2023/2024 due to the actual emissions in 2023/2024 may have been recovered from the pandemic impacts which were on 2020/2021 emissions. The performance for $PM_{2.5}$ total mass and the components are mixed by species and by season, with better performance for some components e.g. nitrate, but worse for total mass and elemental carbon (EC) (Table S4, Figure S1, Table S8). Reasons responsible for the bias in total $PM_{2.5}$ and EC species are tied to missing biomass burning emissions and the pandemic-influenced anthropogenic emissions inventories being used for the post-pandemic years.

Table S4 Annual model performance for species other than ozone and NO_2.

Period	Variable	Unit	#Sites	#Pairs	Obs*	Sim*	NMB(%)	NME(%)	r
2023	24-hr PM_{25}_NH4	ug/m^3	276	28417	0.31	0.23	-25.63	102.27	0.39
	24-hr PM_{25}_EC	ug/m^3	266	27488	0.41	0.2	-51.3	131.67	0.398
	24-hr PM_{25}_OC	ug/m^3	266	27488	1.75	1.21	-30.76	92.76	0.155
	24-hr PM_{25}_NO3	ug/m^3	276	28498	0.62	0.52	-15.92	88.87	0.547
	24-hr PM_{25}_SO4	ug/m^3	276	28650	0.7	0.51	-27.03	65.83	0.497
	24-hr PM_{25}_TOT	ug/m^3	1270	542164	8.5	5.44	-35.97	85.66	0.185
	24-hr PM_{10}	ug/m^3	619	176396	21.02	7.77	-63.04	182.93	0.155
	24-hr PM_{25}_88101	ug/m^3	986	447754	8.75	5.66	-35.29	83.41	0.181
	24-hr PM_{25}_88502	ug/m^3	384	96791	7.29	4.43	-39.2	98.34	0.172
	24-hr Ethylene	ppb	166	12579	0.57	0.57	0.06	106.34	0.254
	24-hr Ethane	ppb	47	6280	7.1	1.99	-71.93	284.57	0.304
	24-hr Acetaldehyde	ppb	114	6555	1.03	0.46	-55.58	142.51	0.211
	1-hr NO	ppb	408	3445163	3.78	0.9	-76.06	388.53	0.178
	1-hr NO_X	ppb	355	2829898	11.14	5.67	-49.07	133.1	0.475
	1-hr CO	ppb	239	1812531	278.57	134.62	-51.68	123.62	0.319
	1-hr SO_2	ppb	389	3127122	1.21	0.53	-56.39	244.06	0.017
	1-hr Isoprene	ppb	30	102827	0.27	0.23	-15.52	99.18	0.51
	1-hr Ethylene	ppb	30	103145	0.94	0.57	-39.65	119.56	0.282
	1-hr Ethane	ppb	29	100898	6.45	1.28	-80.2	406.18	0.444
	1-hr Toluene	ppb	34	136899	0.36	0.32	-10.36	82.06	0.326
	1-hr benzene	ppb	34	135994	0.19	0.12	-38.53	116.75	0.217
	1-hr PM_{25}_TOT	ug/m^3	1049	11042576	8.59	5.42	-36.9	101.06	0.155
	1-hr PM_{25}_88101	ug/m^3	844	9156230	8.75	5.58	-36.28	98.7	0.159
	1-hr PM_{25}_88502	ug/m^3	236	1888805	7.82	4.67	-40.22	114.62	0.13
	1-hr PM_{10}	ug/m^3	451	3483338	22.29	7.87	-64.68	202.65	0.094
2024	24-hr PM_{25}_NH4	ug/m^3	275	26209	0.26	0.2	-24.26	103.49	0.349
	24-hr PM_{25}_EC	ug/m^3	265	25071	0.35	0.2	-41.7	102.56	0.469
	24-hr PM_{25}_OC	ug/m^3	265	25288	1.49	1.26	-15.24	69.62	0.231
	24-hr PM_{25}_NO3	ug/m^3	276	26325	0.59	0.42	-28.74	98.55	0.517
	24-hr PM_{25}_SO4	ug/m^3	276	26324	0.65	0.55	-16.45	57.88	0.448
	24-hr PM_{25}_TOT	ug/m^3	1276	470799	7.1	5.3	-25.3	67.03	0.327
	24-hr PM_{10}	ug/m^3	611	184697	21.17	8.21	-61.22	172.39	0.195
	24-hr PM_{25}_88101	ug/m^3	1005	387767	7.24	5.54	-23.55	63.5	0.349
	24-hr PM_{25}_88502	ug/m^3	359	84756	6.42	4.24	-33.98	87.91	0.235
	24-hr Ethylene	ppb	148	13322	0.68	0.57	-15.52	112.63	0.223
	24-hr Ethane	ppb	53	8066	6.6	1.71	-74.09	302.89	0.305
	24-hr Acetaldehyde	ppb	117	6439	0.92	0.47	-49.17	112.05	0.415
	1-hr NO	ppb	415	3358730	3.76	0.92	-75.67	384.82	0.152
	1-hr NO_X	ppb	363	2746517	10.83	5.8	-46.42	126.78	0.506
	1-hr CO	ppb	229	1662998	273.84	134.62	-50.84	119.76	0.354
	1-hr SO_2	ppb	375	2954709	0.68	0.52	-22.46	148.94	0.105
	1-hr Isoprene	ppb	37	136706	0.27	0.24	-11.48	103.62	0.476
	1-hr Ethylene	ppb	37	136662	0.96	0.53	-44.8	131.62	0.29

1-hr Ethane	ppb	36	135401	6.06	1.23	-79.64	393.51	0.408
1-hr Toluene	ppb	42	160700	0.35	0.31	-11.46	84.21	0.27
1-hr benzene	ppb	43	165574	0.17	0.11	-33.52	105.25	0.31
1-hr PM_{25}_TOT	ug/m^3	1082	9500645	7.1	5.27	-25.77	82.6	0.246
1-hr PM_{25}_88101	ug/m^3	903	7844429	7.17	5.45	-24.02	78.75	0.27
1-hr PM_{25}_88502	ug/m^3	208	1656216	6.78	4.44	-34.52	104.95	0.161
1-hr PM10	ug/m^3	460	3790312	22.22	8.14	-63.34	192.88	0.135

*Annual average.

**Measurements are from the routing monitoring networks, e.g., the nationwide networks SLAMS, PAMS, NCore, CSN, and IMPROVE. The SLAMS network provides hourly measurements of the criteria air pollutants including $PM_{2.5}$, PM_{10}, O_3, SO_2, NO_2, CO etc., typically at the hourly level. The PAMS network measures photochemical smog related species such as O_3, NO, NO_2, NO_y, Total NMOC, and 60 PAMS target VOC compounds in non-attainment areas, again typically at the hourly level. NCore provides enhanced gaseous and particulate measurements. Both CSN and IMPROVE provide speciated $PM_{2.5}$ measurements.

*** 88101 and 88502 are EPA AQS $PM_{2.5}$ parameter codes. 24-hr PM25_88101 data includes measurements from Federal Reference Methods (FRM) and approved Federal Equivalent Methods (FEM) and are generally considered regulatory-grade $PM_{2.5}$ measurements. While 24-hr PM25_88502 data are identified by EPA as "FRM-like" measurements that do not meet all requirements needed for regulatory use and are primarily intended for AQI reporting, forecasting, and research applications rather than NAAQS compliance.

Table S5 Monthly MDA8 O_3 model performance

Period		#Sites	#Pairs	Obs* (ppb)	Sim* (ppb)	NMB(%)	NME(%)	r
2023	Jan	735	20980	34.19	32.59	-4.67	15.47	0.627
	Feb	763	19844	39.44	35.53	-9.91	17.19	0.609
	Mar	1166	35040	43.55	38.83	-10.84	17.93	0.471
	Apr	1198	35221	48.33	39.79	-17.67	24.75	0.56
	May	1209	37181	49.37	38.86	-21.3	30.12	0.574
	Jun	1206	35846	50.88	40.47	-20.46	30.36	0.623
	Jul	1208	36678	47.82	43.92	-8.15	17.7	0.687
	Aug	1201	36364	45.44	44.24	-2.63	14.91	0.696
	Sep	1200	35146	43.47	42.81	-1.51	15.02	0.683
	Oct	1161	34157	38.1	36.69	-3.69	14.88	0.775
	Nov	814	22218	35.7	33.44	-6.34	16.01	0.673
	Dec	787	21535	31.32	31.29	-0.1	16.07	0.599
2024	Jan	775	21746	32.42	32.3	-0.37	15.09	0.597
	Feb	837	21930	38.7	34.85	-9.95	17.52	0.544
	Mar	1142	34344	41.68	37.96	-8.91	16	0.554
	Apr	1164	34572	46.86	39.16	-16.43	23	0.486
	May	1189	36419	47.45	39.18	-17.44	25.26	0.576
	Jun	1194	35050	48.09	40.8	-15.14	23.01	0.673
	Jul	1193	36010	48.21	42.79	-11.24	20.93	0.72

	Aug	1191	36327	46.15	44.12	-4.41	15.21	0.742
	Sep	1189	34842	43.64	41.78	-4.26	16.22	0.733
	Oct	1140	33219	43.89	39.21	-10.68	17.72	0.741
	Nov	841	22341	34.61	32.7	-5.52	16.26	0.632
	Dec	782	21603	32.49	31.79	-2.14	16.35	0.624

*Monthly average.

Table S6 Monthly 1-hr NO_2 model performance

Period		#Sites	#Pairs	Obs* (ppb)	Sim* (ppb)	NMB(%)	NME(%)	r
2023	Jan	427	298194	9.67	6.39	-33.85	83.59	0.595
	Feb	431	276654	9.63	5.55	-42.33	102.78	0.582
	Mar	434	306092	7.28	4.03	-44.62	112.23	0.553
	Apr	434	298921	6.71	3.89	-42.03	107.42	0.559
	May	433	306402	5.95	3.71	-37.57	100.17	0.523
	Jun	436	300296	5.73	3.51	-38.74	104.38	0.489
	Jul	435	303319	5.73	3.78	-34.06	93.35	0.512
	Aug	437	304190	5.85	3.96	-32.31	89.61	0.524
	Sep	432	294030	6.51	4.71	-27.64	80.87	0.56
	Oct	430	299808	8.06	5.46	-32.31	82.23	0.625
	Nov	428	292228	10.25	6.19	-39.63	92.19	0.628
	Dec	428	302292	10.64	7.28	-31.52	77.23	0.616
2024	Jan	436	308120	9.85	6.65	-32.43	80.25	0.608
	Feb	437	288809	8.92	5.61	-37.11	90.17	0.609
	Mar	422	300890	6.73	4.17	-38.14	98.4	0.562
	Apr	422	287897	6.2	3.77	-39.29	101.97	0.571
	May	418	295272	5.49	3.68	-33.03	94.7	0.5
	Jun	423	287369	5.41	3.49	-35.52	100.12	0.468
	Jul	421	295985	5.77	3.86	-33.13	95.3	0.471
	Aug	424	297844	6	4.22	-29.66	87.97	0.512
	Sep	417	287054	6.77	4.92	-27.36	82.44	0.564
	Oct	418	296900	9.01	6.02	-33.13	85.22	0.599
	Nov	420	289289	8.92	5.78	-35.2	87.12	0.613
	Dec	423	298199	10.7	7.16	-33.1	82.3	0.593

*Monthly average.

Table S7 Monthly 24-hr benzene model performance

Period		#Sites	#Pairs	Obs* (ppb)	Sim* (ppb)	NMB(%)	NME(%)	r
2023	Jan	162	1064	0.24	0.16	-32.31	75.77	0.306
	Feb	155	908	0.21	0.14	-34.23	76.83	0.463
	Mar	154	1206	0.16	0.1	-34.57	77.93	0.348
	Apr	151	1091	0.14	0.09	-37.98	93.26	0.314
	May	154	1139	0.14	0.09	-37.21	96.3	0.34
	Jun	156	1504	0.17	0.07	-58	166.35	0.185
	Jul	156	1532	0.16	0.08	-51.04	134.72	0.053
	Aug	156	1655	0.17	0.08	-53.48	140.23	0.113
	Sep	146	1160	0.18	0.1	-45.21	112.71	0.338
	Oct	150	1145	0.21	0.12	-43.64	104.24	0.318
	Nov	148	1069	0.24	0.15	-35.2	84.57	0.285
	Dec	146	1100	0.27	0.19	-29.24	74.18	0.247
2024	Jan	139	1201	0.24	0.18	-24.02	64.92	0.277
	Feb	138	954	0.21	0.16	-24.01	65.2	0.394
	Mar	141	1257	0.19	0.11	-40.73	100.75	0.057
	Apr	140	1090	0.13	0.09	-33.18	85.7	0.31
	May	143	1432	0.13	0.08	-39.61	97.11	0.269
	Jun	155	1779	0.12	0.07	-38.52	94.77	0.364
	Jul	150	1699	0.13	0.08	-43.2	101.27	0.292
	Aug	146	1587	0.16	0.09	-46.93	109.43	0.344
	Sep	136	1121	0.18	0.1	-42.36	102.58	0.312
	Oct	127	1061	0.24	0.13	-43.74	111.47	0.22
	Nov	125	1085	0.22	0.14	-38.14	96.51	0.219
	Dec	125	1043	0.26	0.18	-30.43	80.51	0.262

*Monthly average.

Table S8 Monthly 24-hr PM_{25} model performance

Period		#Sites	#Pairs	Obs* (ug/m^3)	Sim* (ug/m^3)	NMB(%)	NME(%)	r
2023	Jan	1224	48633	7.6	7.06	-7.05	55.56	0.476
	Feb	1224	44363	7.53	6.5	-13.72	57.46	0.368
	Mar	1221	49706	6.81	4.94	-27.42	67.46	0.386
	Apr	1229	48316	6.76	4.23	-37.42	75.4	0.494
	May	1228	49862	8.03	4.29	-46.58	100.62	0.199
	Jun	1227	48171	14.25	4.15	-70.9	251.59	0.097
	Jul	1225	45803	10.24	4.57	-55.4	131.99	0.272
	Aug	1235	44160	9.88	4.65	-53	122.27	0.17
	Sep	1233	41972	8.12	5.28	-34.98	72.59	0.234
	Oct	1236	41622	6.6	5.79	-12.33	51.03	0.437
	Nov	1234	39519	8.06	7.21	-10.56	51.22	0.344
	Dec	1232	40037	7.84	7.35	-6.34	60.26	0.395
2024	Jan	1236	40430	6.89	6.73	-2.29	58.29	0.334
	Feb	1236	37777	6.8	6.63	-2.44	55.51	0.417
	Mar	1226	40418	5.72	4.61	-19.31	59.86	0.396
	Apr	1224	38835	5.77	3.97	-31.2	66.05	0.466
	May	1221	39900	6.35	4.04	-36.27	72.86	0.555
	Jun	1225	38664	6.9	4.06	-41.08	79.95	0.512
	Jul	1224	39699	9.17	4.32	-52.91	121.12	0.187
	Aug	1223	40312	8.64	4.88	-43.46	89.7	0.272
	Sep	1225	38705	6.92	5.23	-24.43	61.03	0.208
	Oct	1226	39673	7.6	6.72	-11.58	50.01	0.391
	Nov	1115	37685	6.41	5.26	-17.93	58.12	0.273
	Dec	1118	38701	7.99	7.24	-9.5	58.37	0.395

*Monthly average.

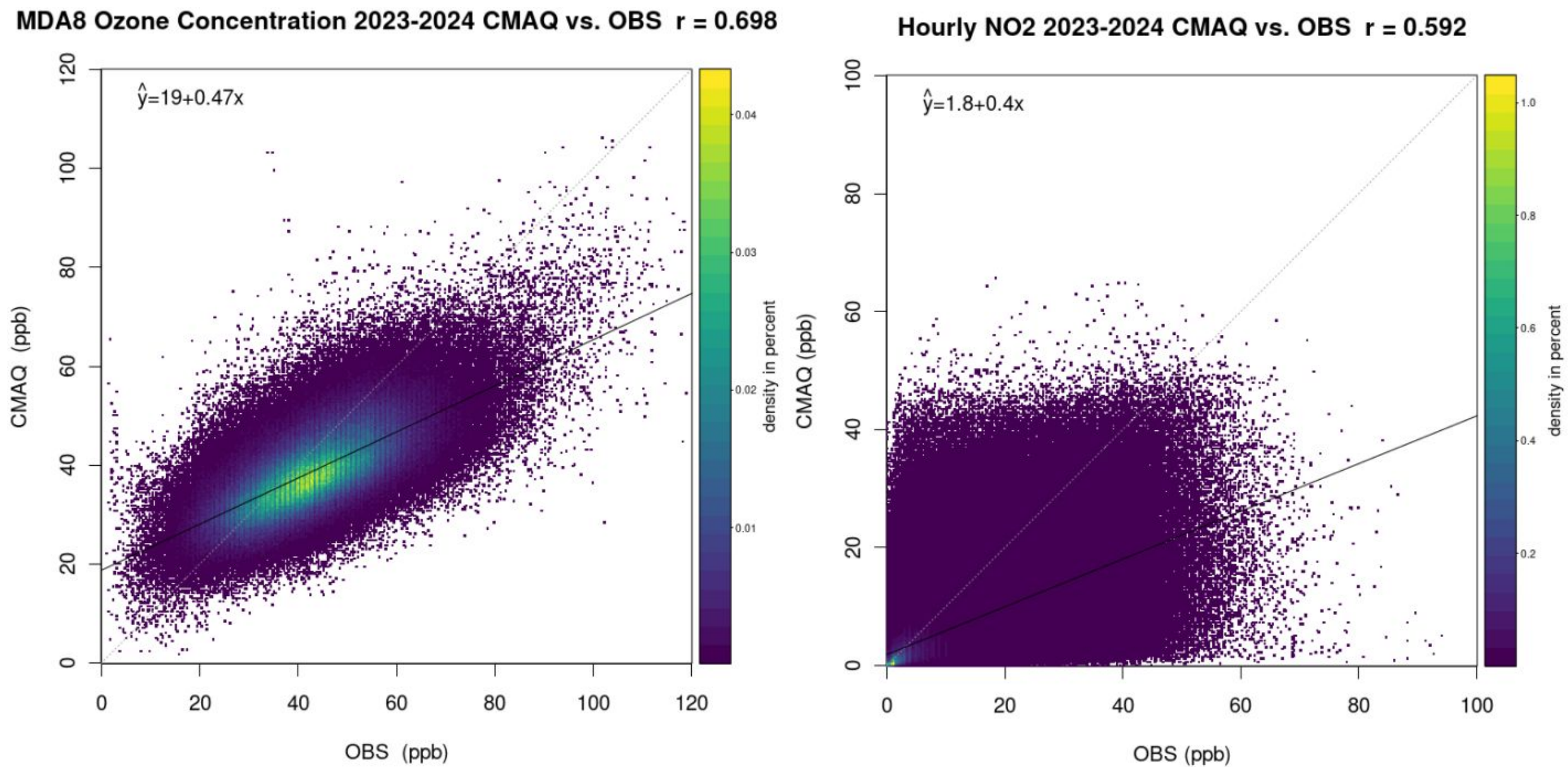
MDA8 Ozone Concentration 2023-2024 CMAQ vs. OBS r = 0.698
ŷ=19+0.47x
CMAQ (ppb)
OBS (ppb)
density in percent
Hourly NO2 2023-2024 CMAQ vs. OBS r = 0.592
ŷ=1.8+0.4x
CMAQ (ppb)
OBS (ppb)
density in percent

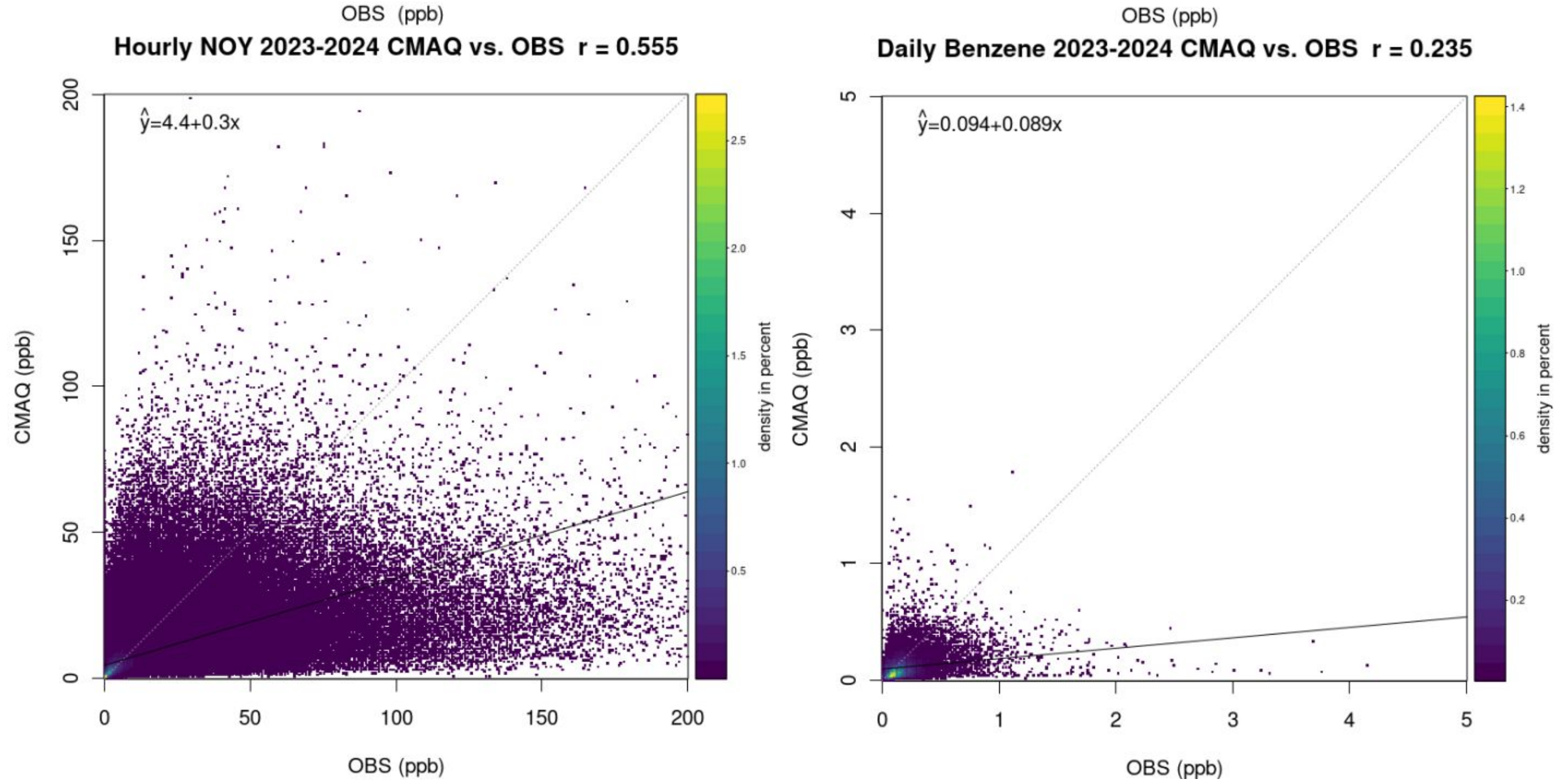
Hourly NOY 2023-2024 CMAQ vs. OBS r = 0.555
ŷ=4.4+0.3x
CMAQ (ppb)
OBS (ppb)
density in percent
Daily Benzene 2023-2024 CMAQ vs. OBS r = 0.235
ŷ=0.094+0.089x
CMAQ (ppb)
OBS (ppb)
density in percent

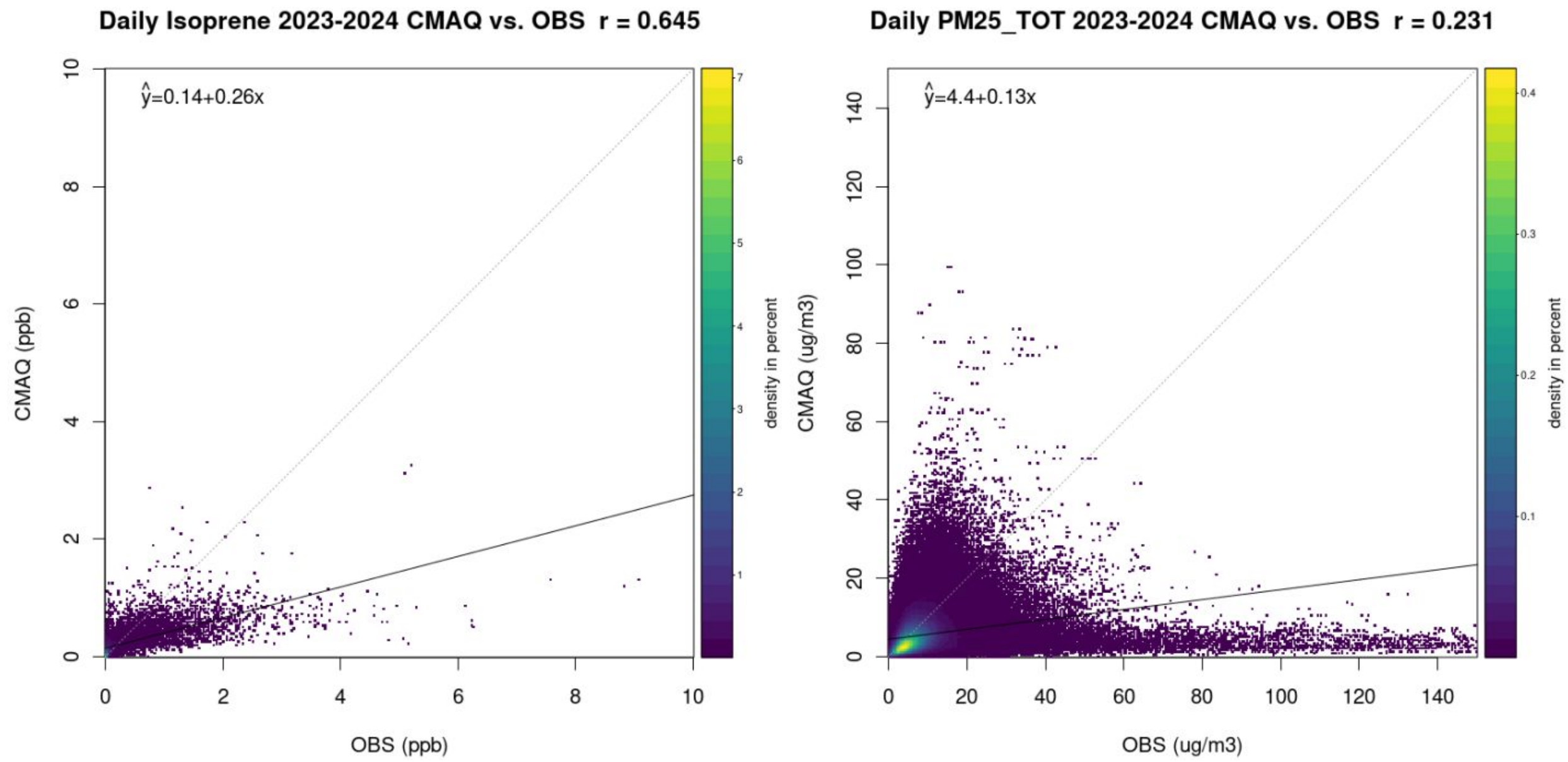

Figure S1. Density scatter plots of MDA8 O_3, 1-hr NO_2, 1-hr NO_y, 24-hr benzene, 24-hr Isoprene, and 24-hr PM_{25}_TOT CMAQ simulations versus observations (OBS).

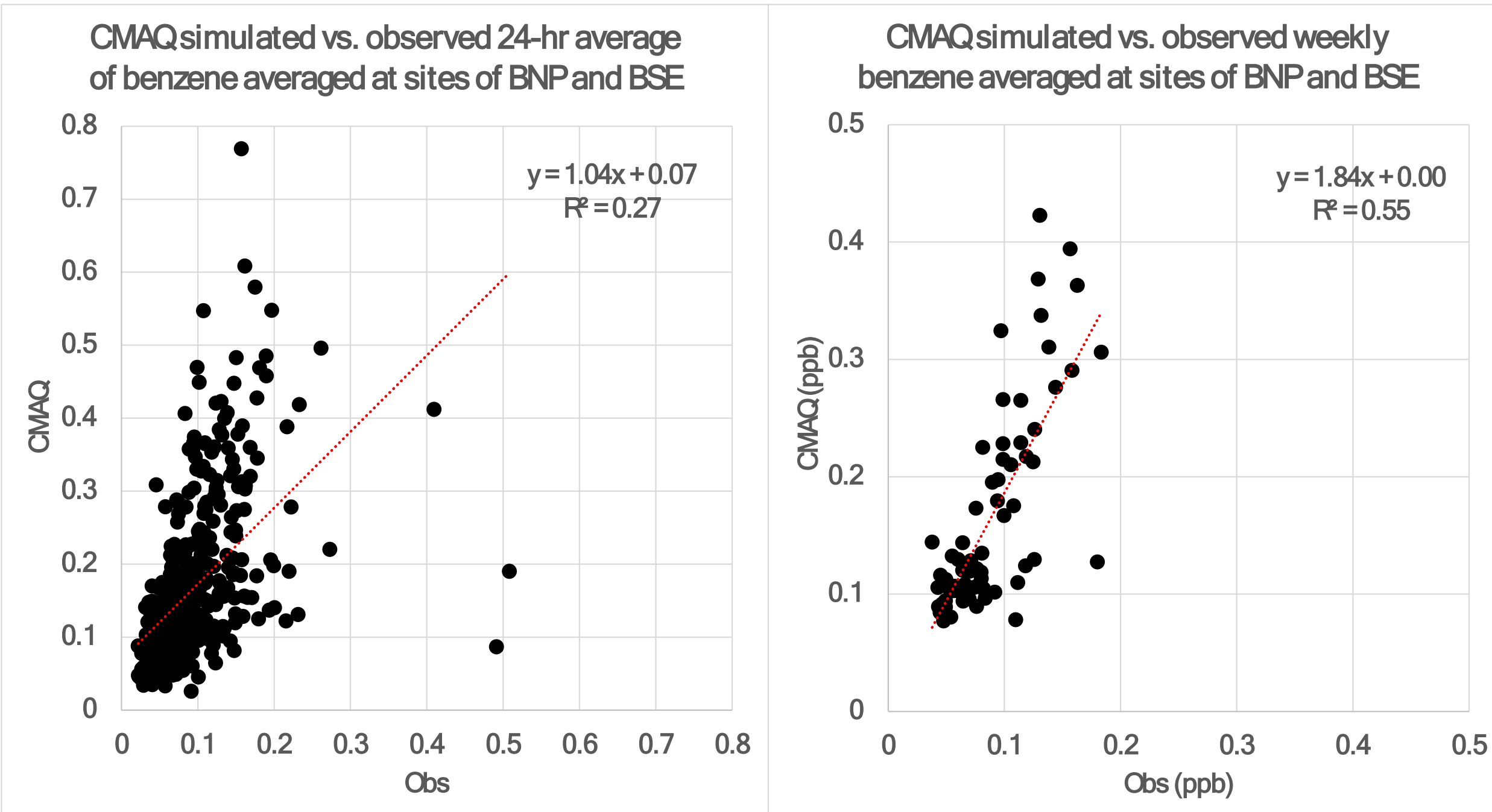

Figure S2. Scatter plots of CMAQ simulated 24-hr benzene (left) and weekly benzene concentrations (right) against averaged observations at the Broomfield North Pecos (BNP) and Broomfield Soaring Eagle (BSE) sites near Boulder, CO (see monitor information on website https://bouldair.com/).

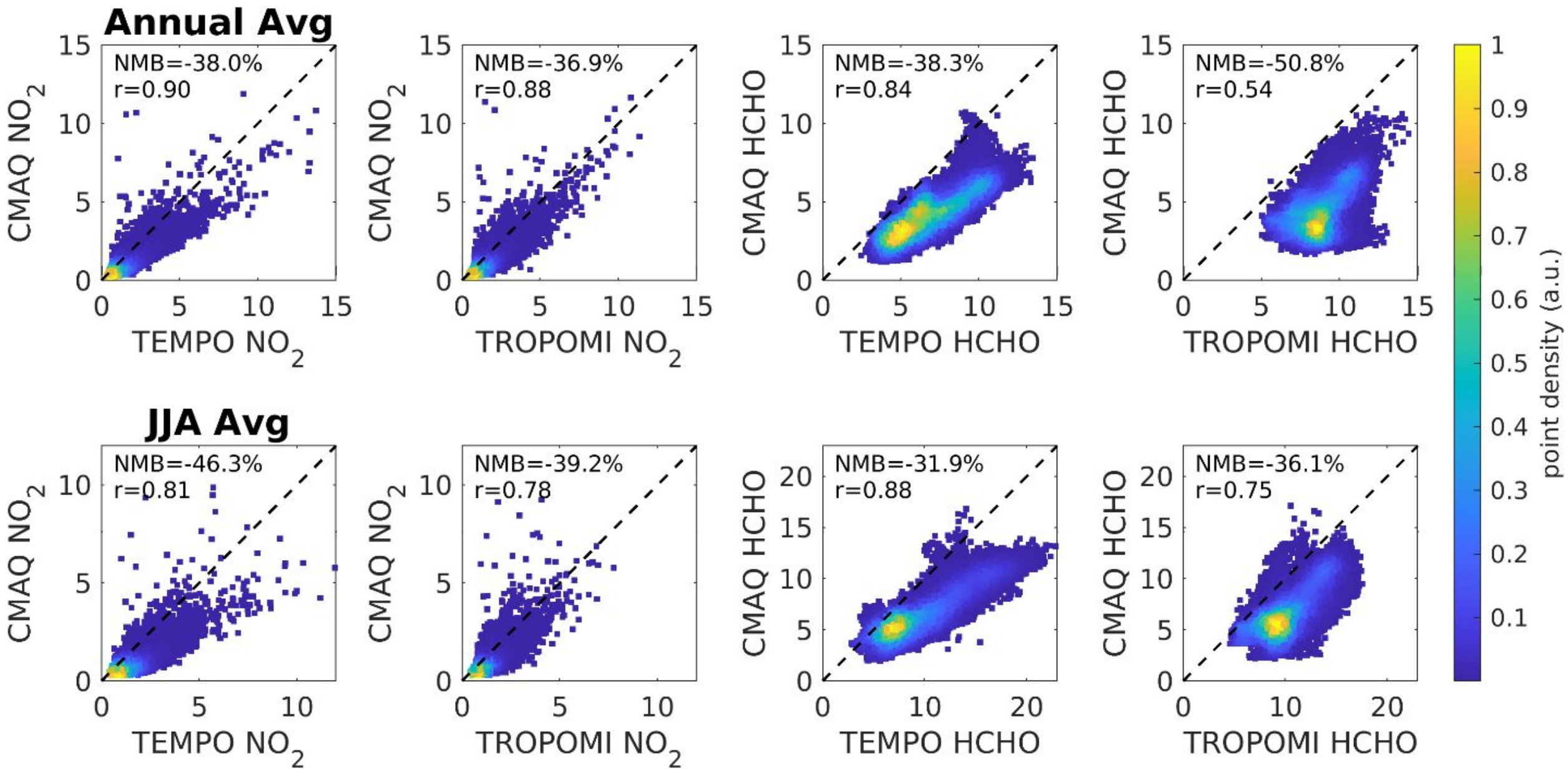

Figure S3. Correlation and bias of CMAQ with satellite-based observations. Each point represents one grid cell in the domain, averaged annual (top row) or over summer months (bottom row). All values are in units of 10^{15} molecules cm^{-2}.

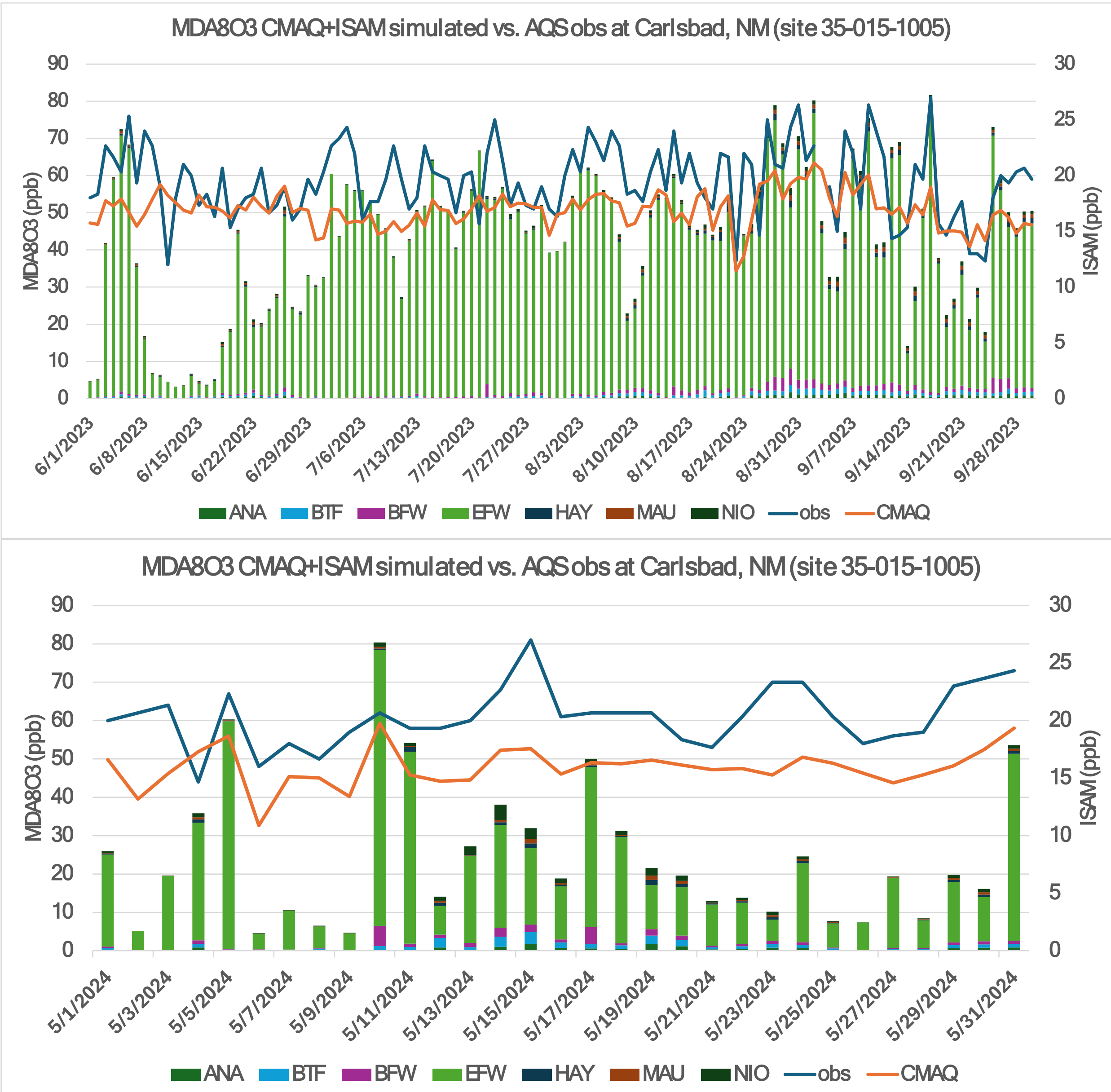

Figure S4. CMAQ simulated timeseries of major shale plays' contributions to MDA8 O_3 with simulated MDA8O_3 concentration against observed at AQS site 35-015-1005 in Carlsbad, Eddy County, New Mexico. The largest contributor is "background" which is not shown to make the other contributions more evident.

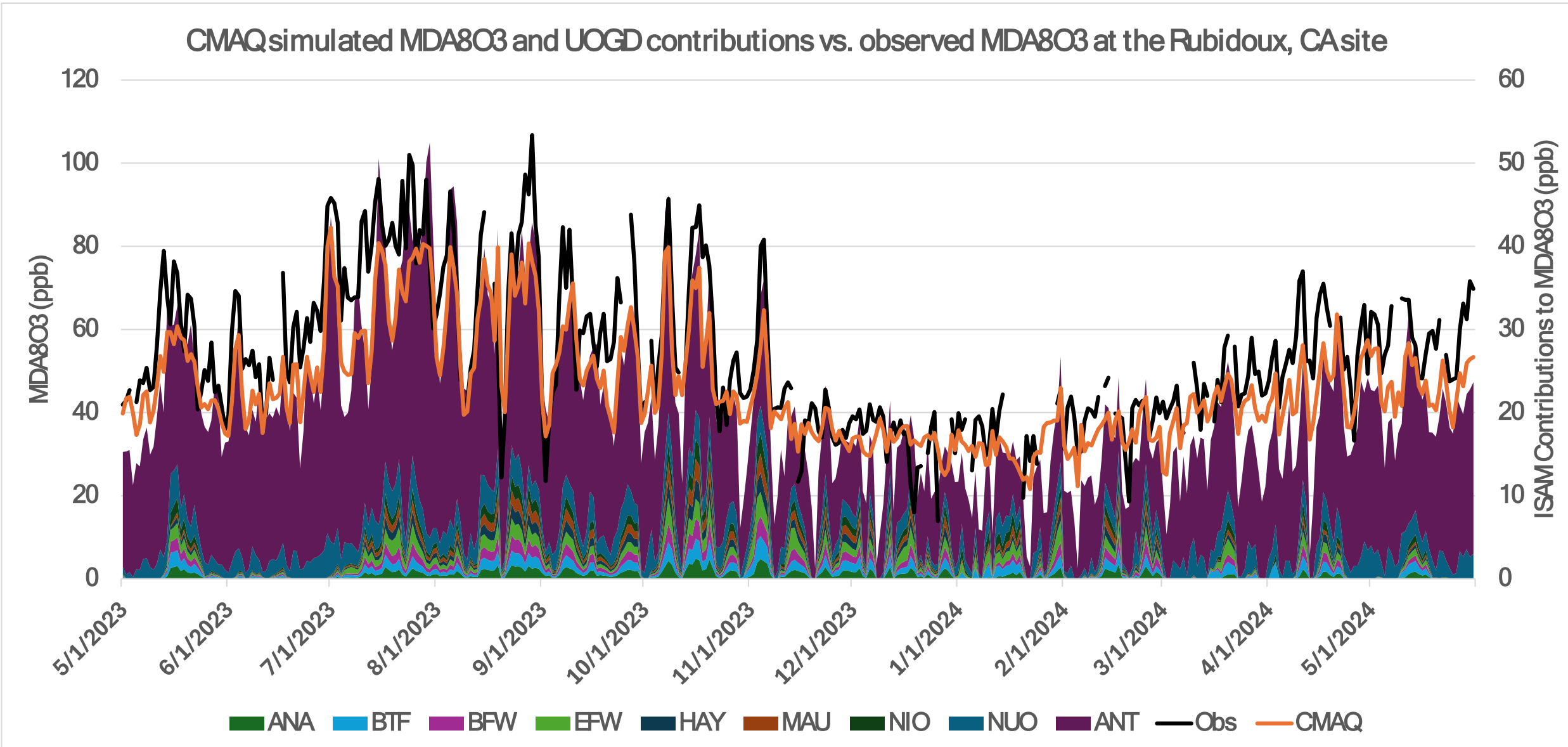

Figure S5. CMAQ simulated timeseries of major shale plays' contributions to MDA8 O_3 with simulated $MDA8O_3$ concentration against observed at AQS site 06-065-8001 in Rubidoux, California. Also shown are contributions from other anthropogenic sectors of NUO (non-UOGD oil and gas) and ANT (other anthropogenic including onroad emissions). The largest contributor is "background" which is not shown to make the other contributions more evident.

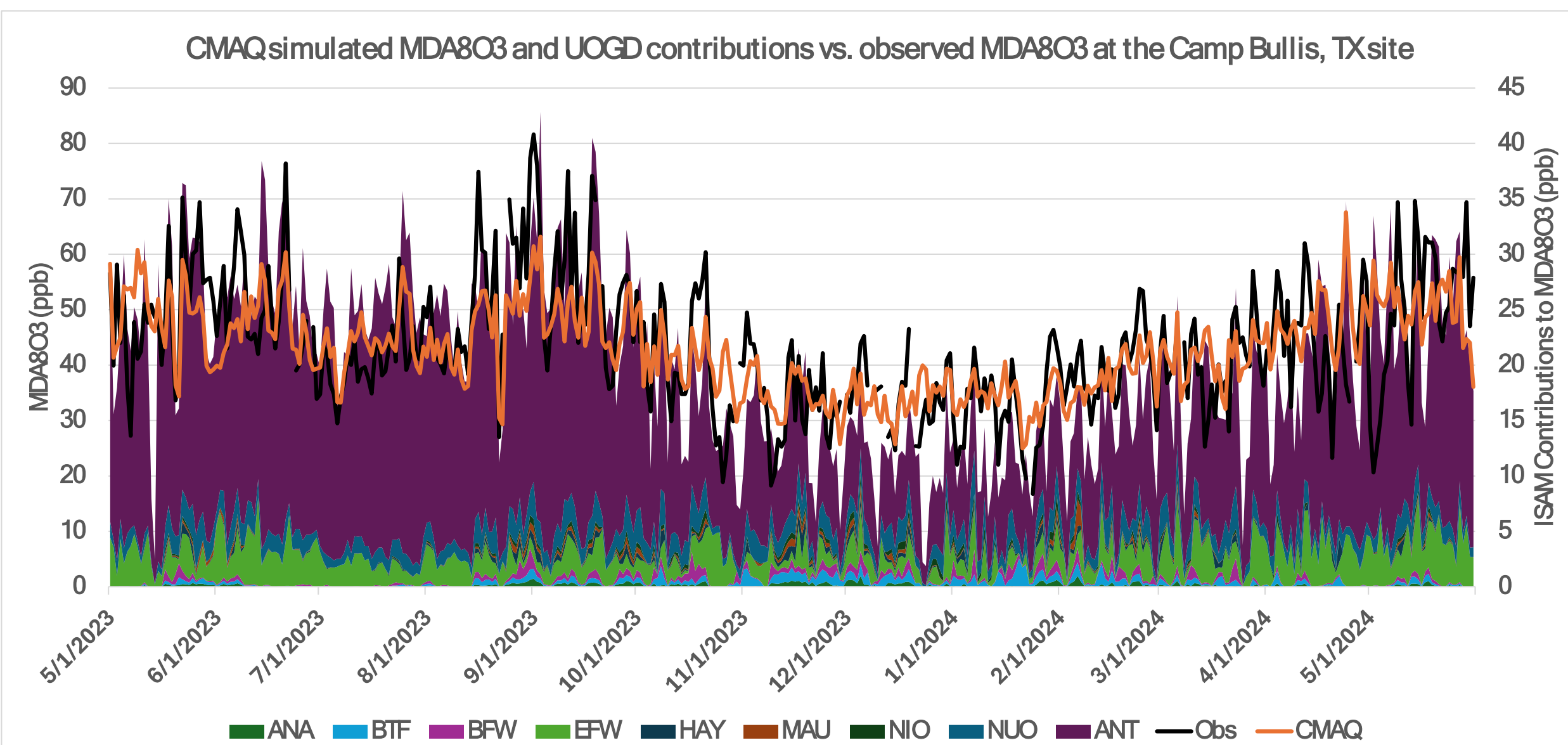

Figure S6. CMAQ simulated timeseries of major shale plays' contributions to MDA8 O_3 with simulated $MDA8O_3$ concentration against observed at AQS site 48-029-0052 in Camp Bullis, Texas. Also shown are contributions from other anthropogenic sectors of NUO (non-UOGD oil and gas) and ANT (other anthropogenic including onroad emissions). The largest contributor is "background" which is not shown to make the other contributions more evident.

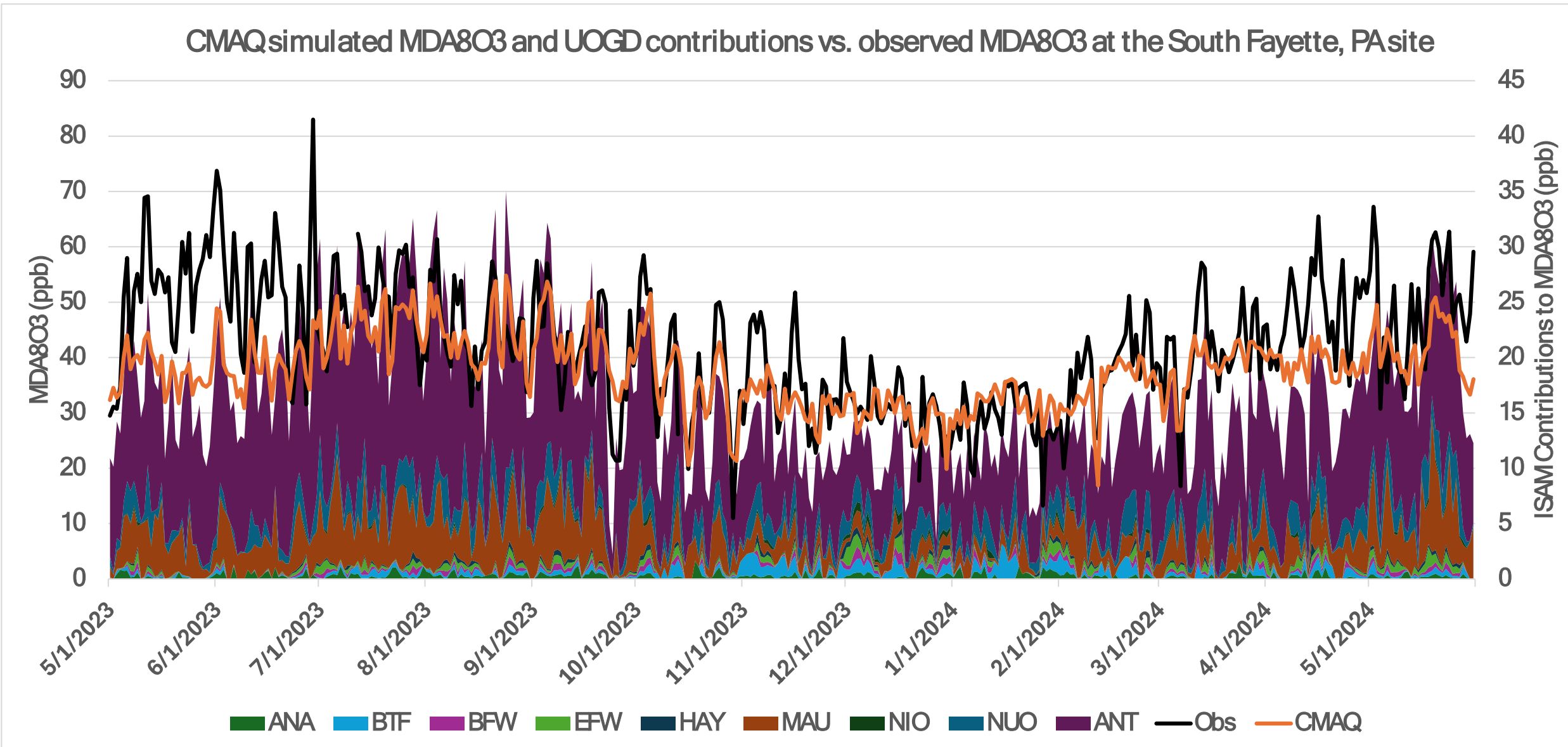

Figure S7. CMAQ simulated timeseries of major shale plays' contributions to MDA8 O_3 with simulated MDA8O_3 concentration against observed at AQS site 42-003-1008 in South Fayette, Pennsylvania. Also shown are contributions from other anthropogenic sectors of NUO (non-UOGD oil and gas) and ANT (other anthropogenic including onroad emissions). The largest contributor is "background" which is not shown to make the other contributions more evident.

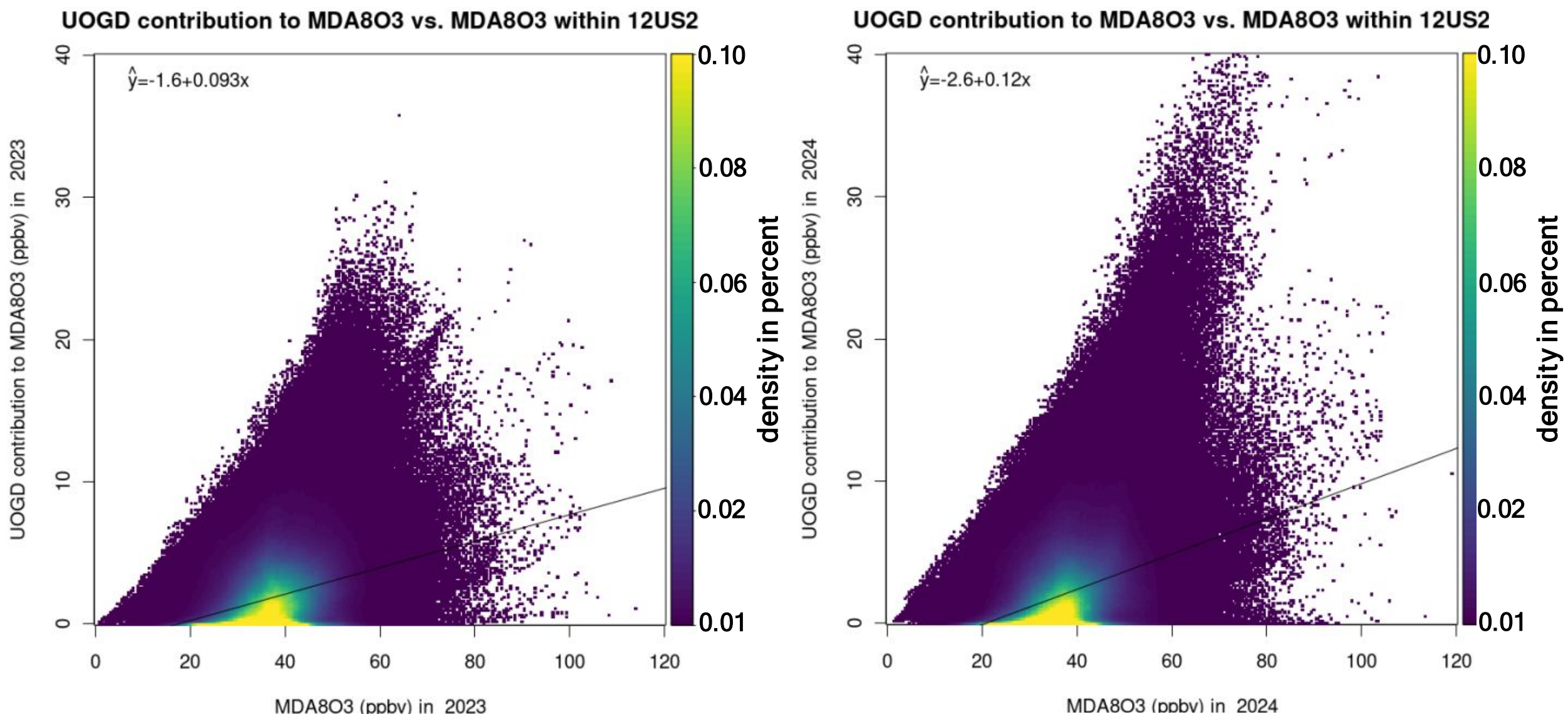

Figure S8. Scatter plot colored by density of CMAQ-ISAM simulated UOGD contributions to MDA8O3 versus MDA8O3 in 2023 (left) and in 2024 (right) at grid cells of 12US2 with the pairs of UOGD contributions less than 0.0001 ppb being removed. Note that the averaged UOGD contribution to MDA8O3 over 70 ppb is 6.26 ppb in 2023 corresponding to MDA8O3 average of 75.2 ppb and is 9.27 ppb in 2024 corresponding to MDA8O3 average of 75.5 ppb.

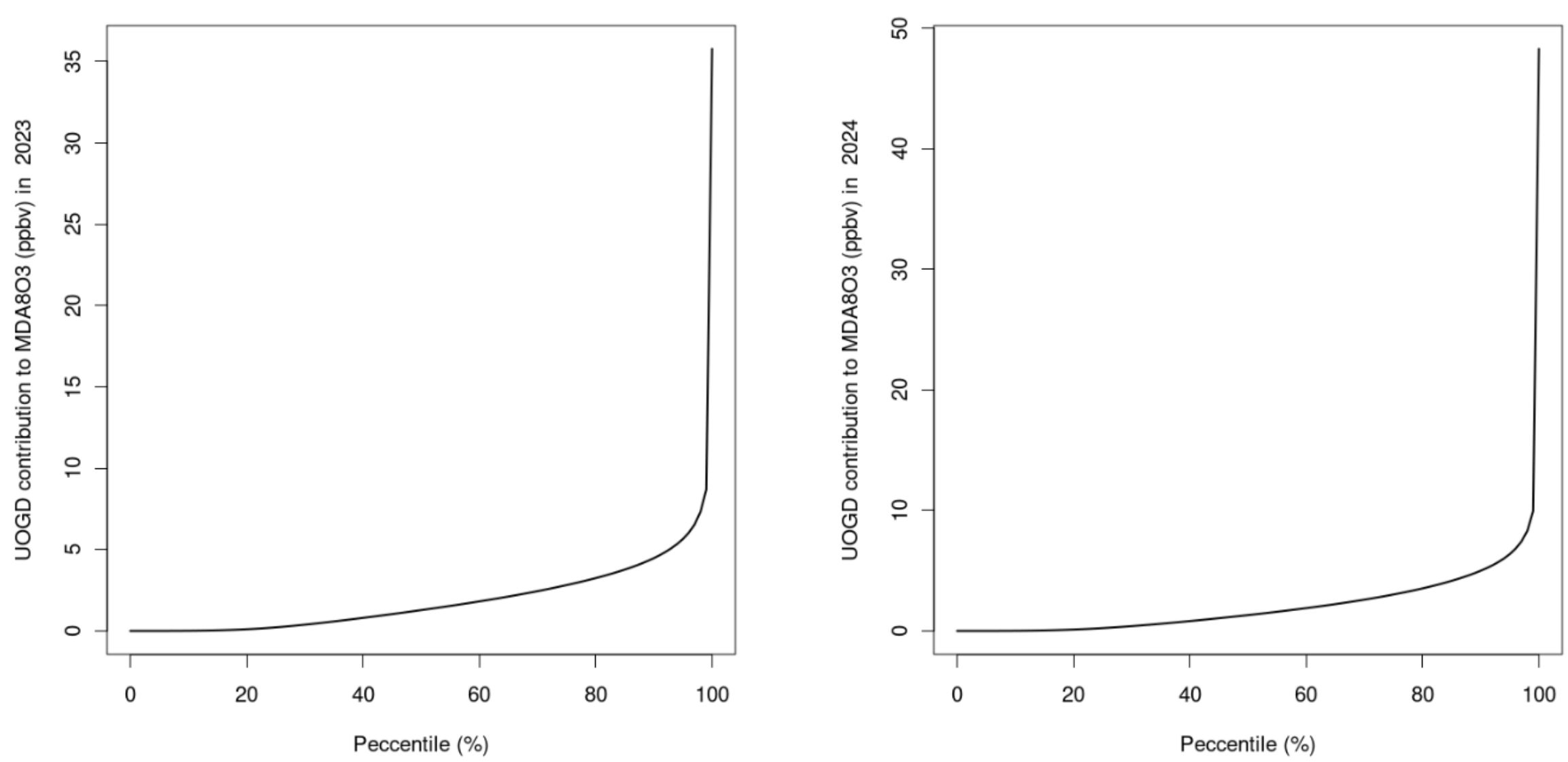

Figure S9. Quantile plot of CMAQ-ISAM simulated UOGD contributions to MDA8O3 in 2023 (left) and in 2024 (right) at grid cells of 12US2 with the data value less than 0.0001 ppb being removed. Note that the 5.0 ppb of UOGD contribution corresponds to the percentile of 92.6% in 2023 and 90.0% in 2024, respectively, while the corresponding UOGD contributions to percentile of 5%, 25%, 50%, 75%, 95% are 0.0015, 0.22, 1.28, 2.82 and 5.66 ppb in 2023 and 0.0016, 0.24, 1.32, 3.03, and 6.38 ppb in 2024, respectively.

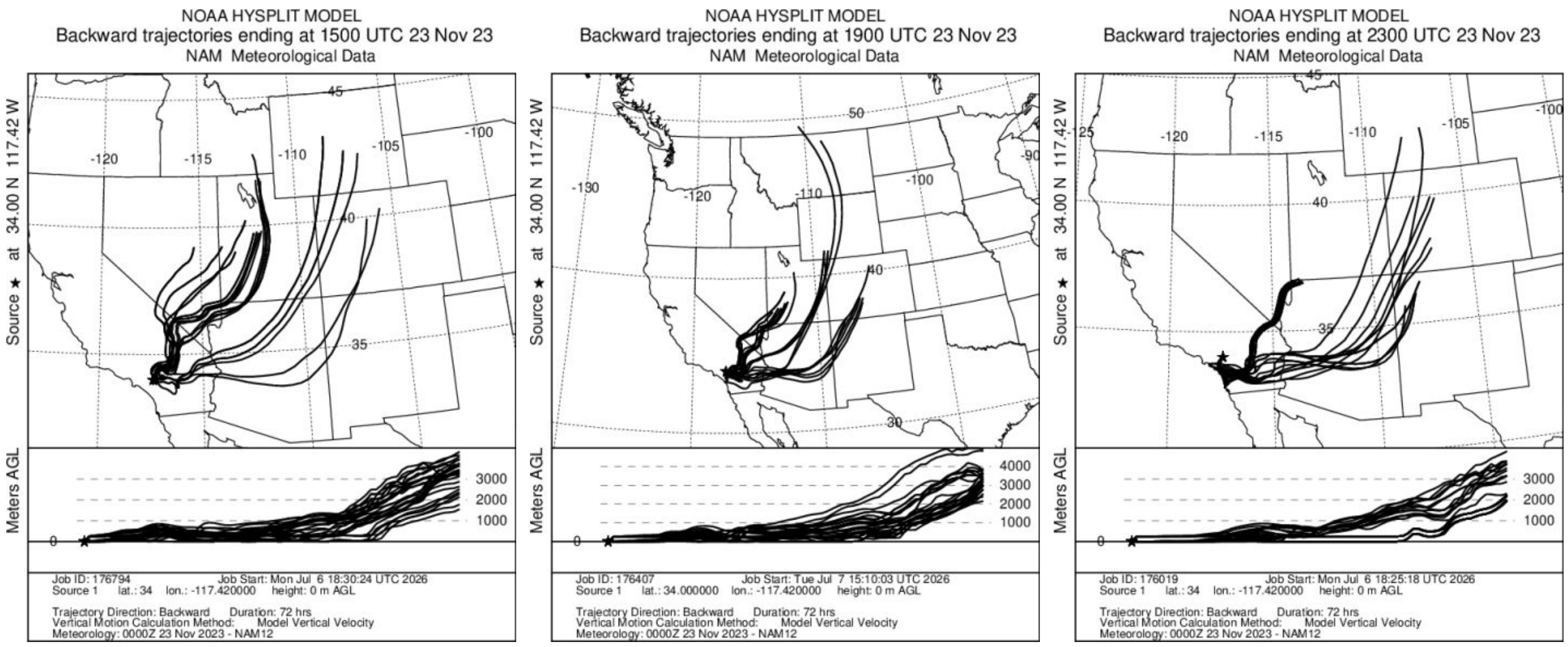

Figure S10. Backward 72 hours trajectories ending at 15, 19 and 23 UTC (8, 12 and 16 PST) on November 23, 2023 from ground level in Rubidoux, California (latitude 34, longitude -117.42) produced by NOAA HYSPLIT model using the NAM 12km meteorological data. It shows transports from Colorado and New Mexico to Southern California.

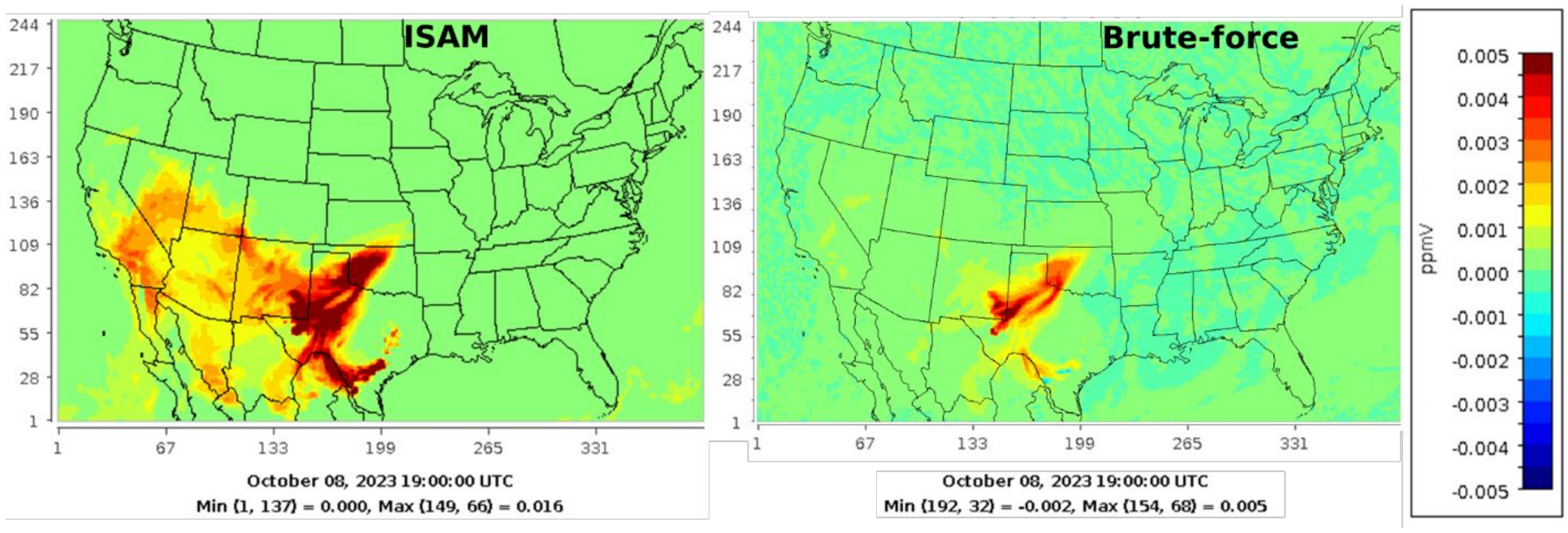

Figure S11a. Snap shots of ISAM (left) and brute-force (right) simulated EFW (shale plays of Eagle Ford and Wolfcamp) contribution to hourly ozone concentration at 19 UTC (12 PST) on October 8, 2023. It shows the two methods that predict EFW impacts reaching Southern California with spatial similarity but differ in magnitude.

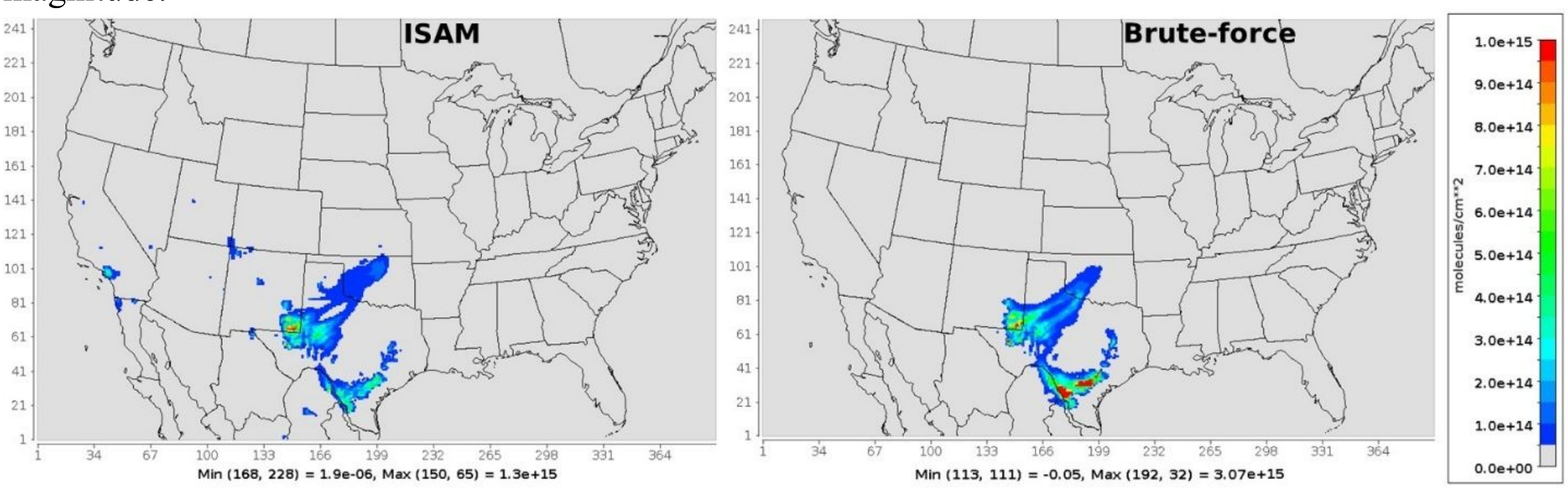

Figure S11b. Snap shots of ISAM (left) and brute-force (right) simulated EFW (shale plays of Eagle Ford and Wolfcamp) contribution to NO2 VCD averaged at 13-14 LST on October 8, 2023.

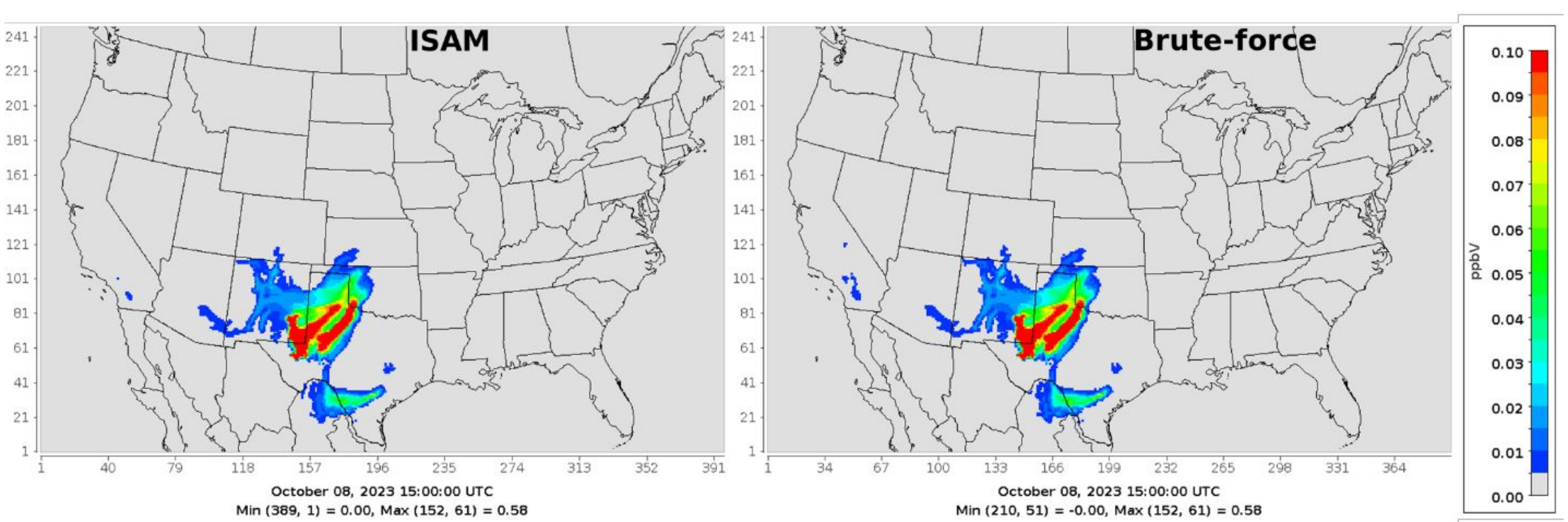

Figure S11c. Snap shots of ISAM (left) and brute-force (right) simulated EFW (shale plays of Eagle Ford and Wolfcamp) contribution to hourly benzene concentration at 15 UTC on October 8, 2023.

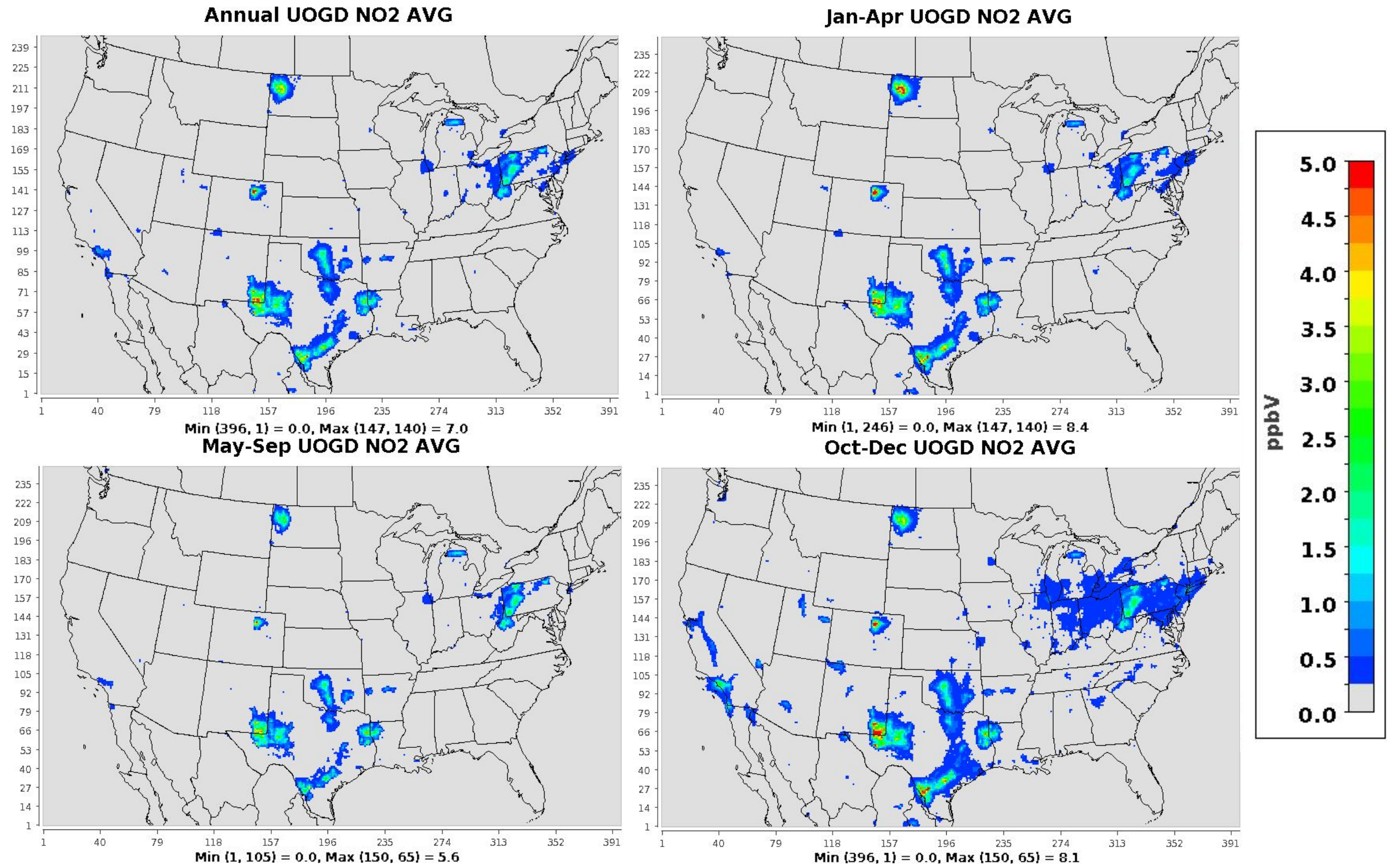

Figure S12a. 2023 annual and seasonal average of surface NO_2 due to UOGD simulated by CMAQ ISAM: annual (top left), Jan-Apr (top right), May-sept (bottom left) and Oct-Dec (bottom right).

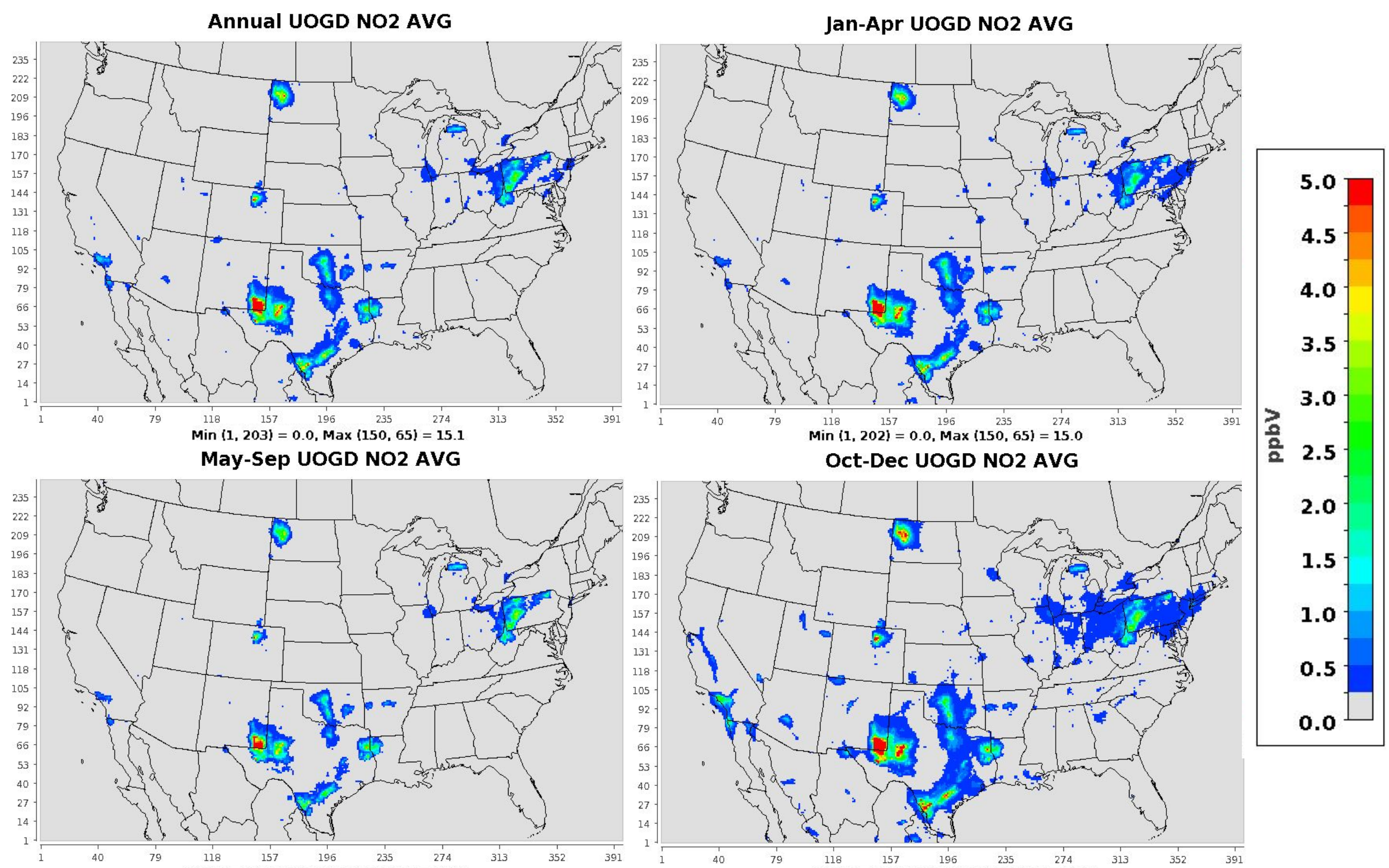

Figure S12b. 2024 annual and seasonal average of surface NO_2 due to UOGD simulated by CMAQ ISAM: annual (top left), Jan-Apr (top right), May-sept (bottom left) and Oct-Dec (bottom right).

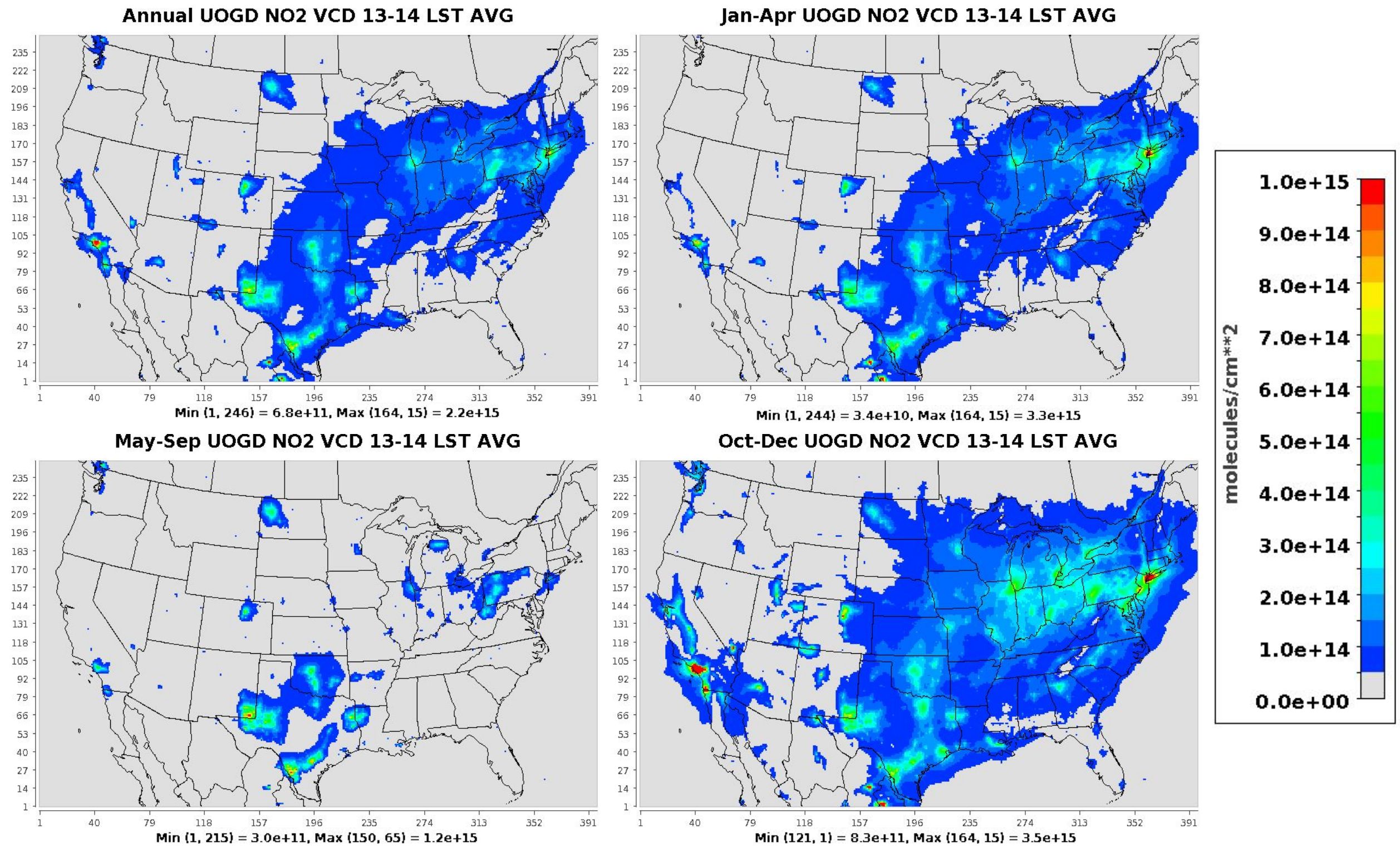

Figure S13a. 2023 annual and seasonal average of NO_2 VCD 13-14 LST AVG due to UOGD simulated by CMAQ ISAM: annual (top left), Jan-Apr (top right), May-sept (bottom left) and Oct-Dec (bottom right). (While the impact of all of the modeled shale plays are shown together here, we have them separately).

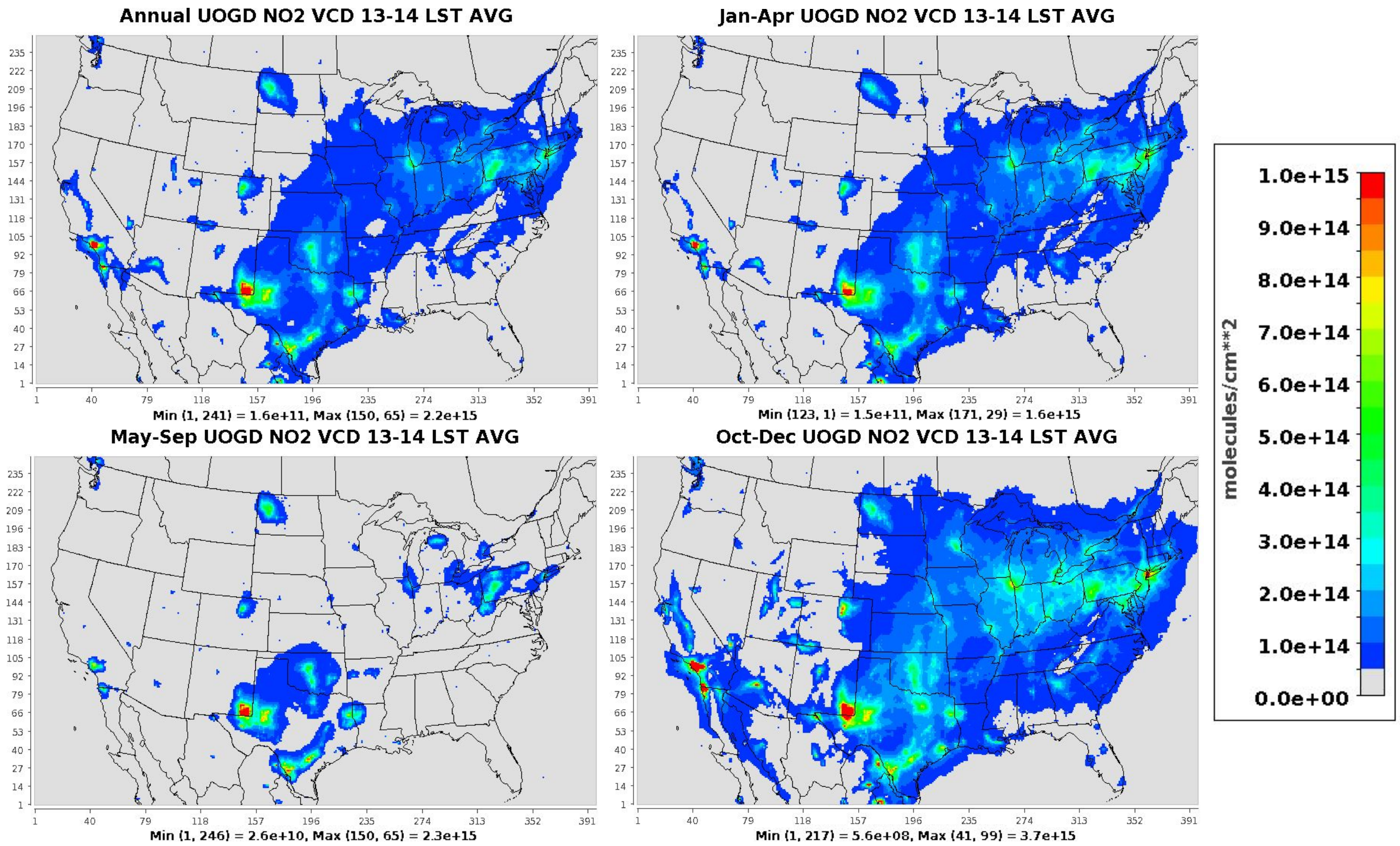

Figure S13b. 2024 annual and seasonal average of NO_2 VCD 13-14 LST AVG due to UOGD simulated by CMAQ ISAM: annual (top left), Jan-Apr (top right), May-sept (bottom left) and Oct-Dec (bottom right). (While the impact of all of the modeled shale plays are shown together here, we have them separately).

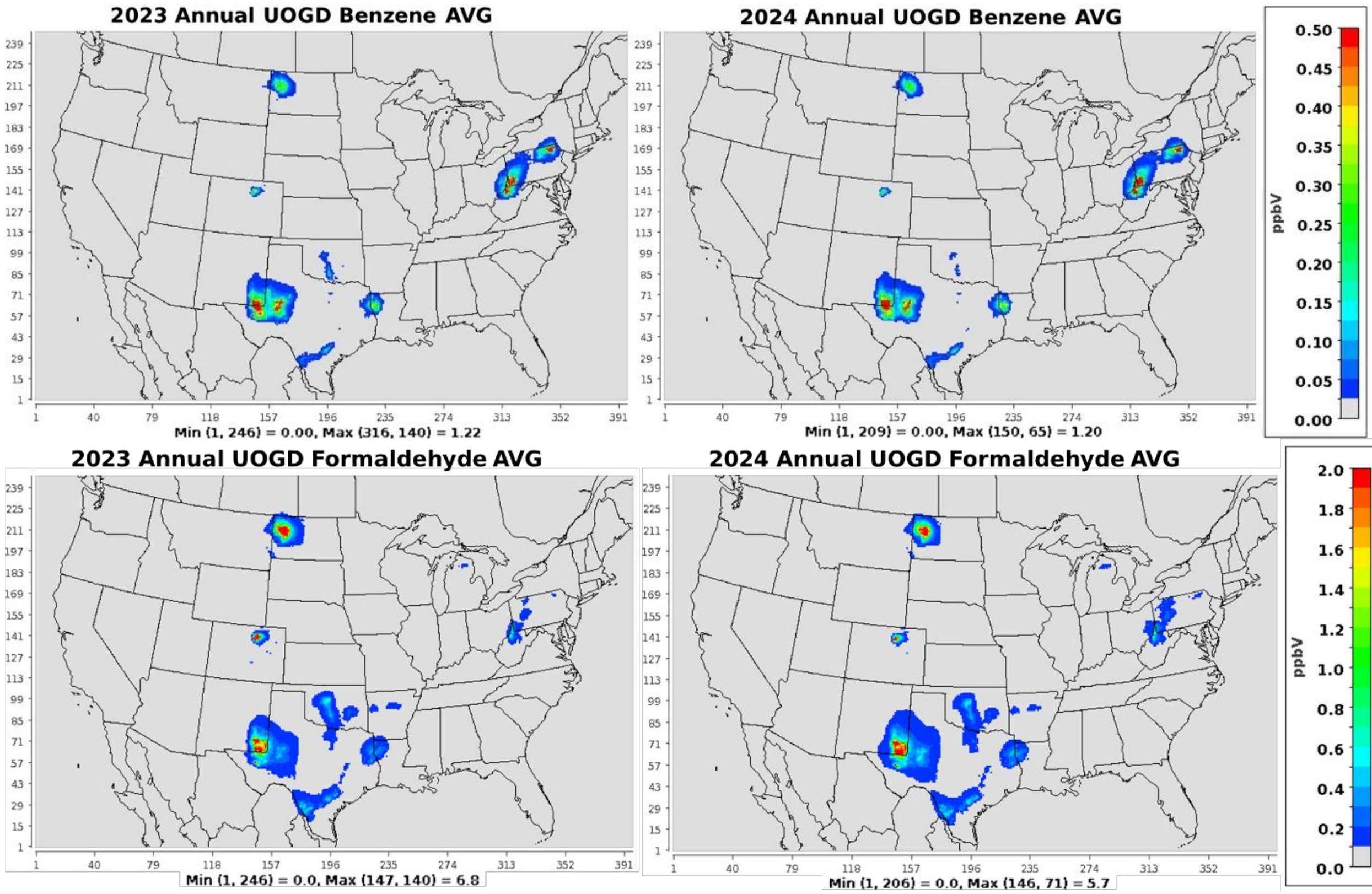

Figure S14. Annual average UOGD contributions to other pollutants simulated by CMAQ ISAM: benzene (top row) and formaldehyde (HCHO, bottom row) in 2023 (left) and 2024 (right).

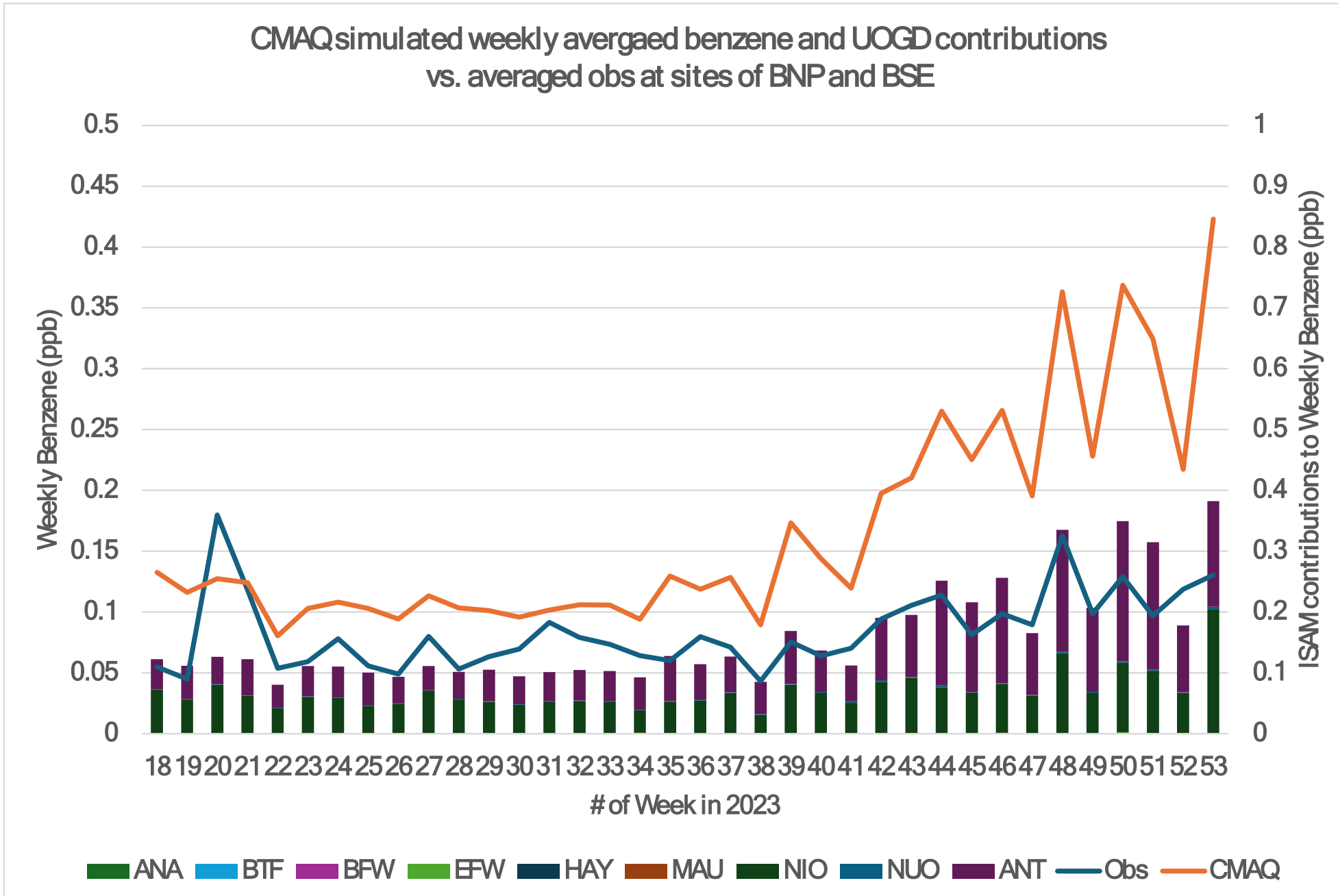

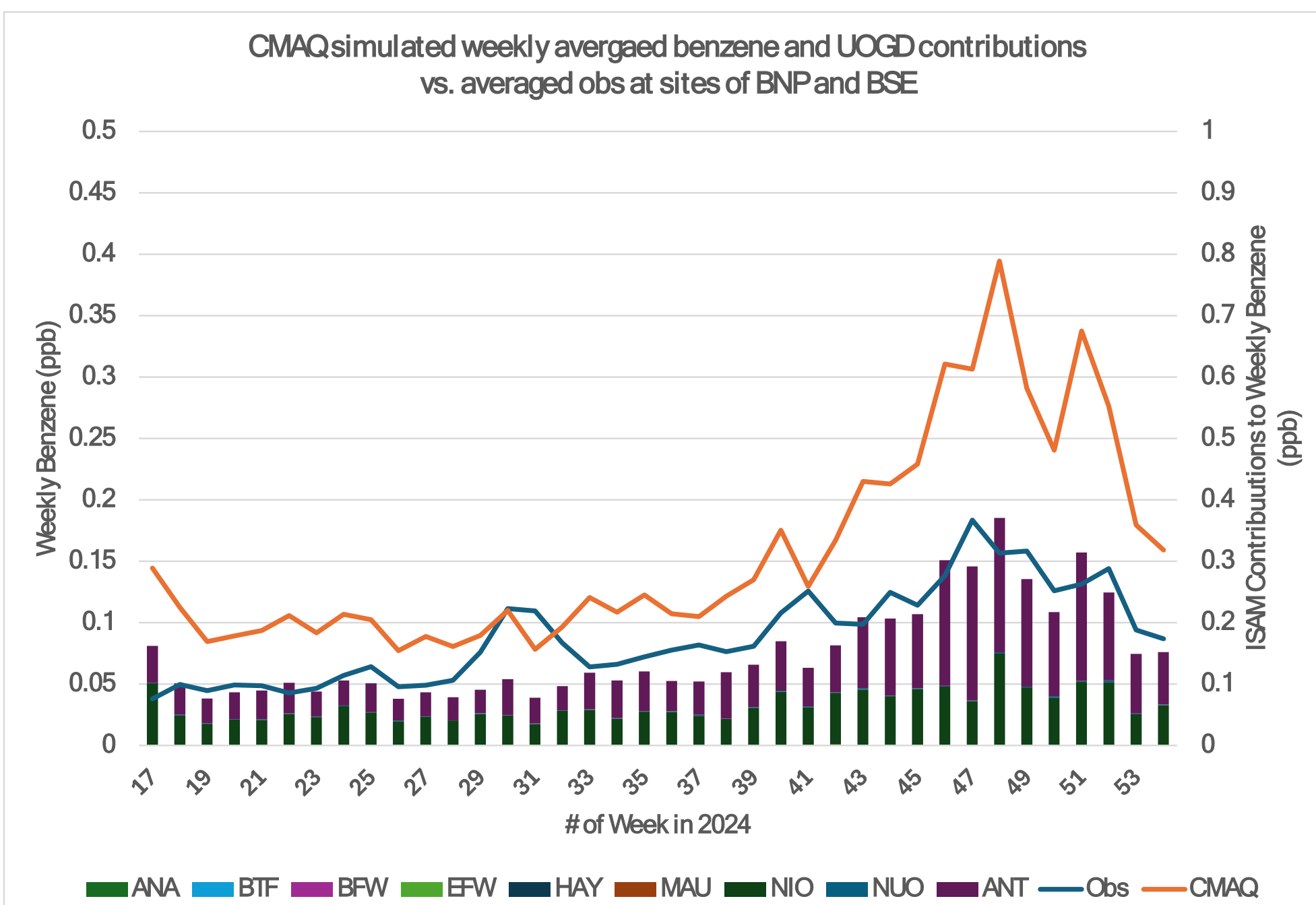

Figure S15. CMAQ simulated timeseries of major shale plays' contributions to weekly averaged benzene concentration with simulated weekly benzene concentration against averaged observations at the Broomfield North Pecos (BNP) and Broomfield Soaring Eagle (BSE) sites near Boulder, CO (see monitor information on website https://bouldair.com/). Also shown are contributions from other anthropogenic sectors of NUO (non-UOGD oil and gas) and ANT (other anthropogenic including onroad emissions).
